# The 2022 Outbreaks of African Swine Fever Virus Demonstrate the First Report of Genotype II in Ghana

**DOI:** 10.3390/v15081722

**Published:** 2023-08-11

**Authors:** Edward Spinard, Ayushi Rai, Jehadi Osei-Bonsu, Vivian O’Donnell, Patrick T. Ababio, Daniel Tawiah-Yingar, Daniel Arthur, Daniel Baah, Elizabeth Ramirez-Medina, Nallely Espinoza, Alyssa Valladares, Bonto Faburay, Aruna Ambagala, Theophlius Odoom, Manuel V. Borca, Douglas P. Gladue

**Affiliations:** 1 U.S. Department of Agriculture, Agricultural Research Service, Foreign Animal Disease Research Unit, Plum Island Animal Disease Center, Orient, NY 11957, USA; edward.spinard@usda.gov (E.S.); elizabeth.ramirez@usda.gov (E.R.-M.); nallely.espinoza@usda.gov (N.E.);; 2 U.S. Department of Agriculture, Agricultural Research Service, Foreign Animal Disease Research Unit, National Bio and Agro-Defense Facility, Unit Name, Manhattan, KS 66502, USA; 3 Center of Excellence for African Swine Fever Genomics, Guilford, CT 06437, USAaruna.ambagala@inspection.gc.ca (A.A.); theodoom@yahoo.com (T.O.); 4 Accra Veterinary Laboratory of Veterinary Services Directorate, Accra P.O. Box GA184, Ghanaginolapaatee@gmail.com (P.T.A.); nanayawtee@icloud.com (D.T.-Y.); danielarthur42681@gmail.com (D.A.); dannyneph@gmail.com (D.B.); 5 Animal and Plant Inspection Service, USDA, Greenport, NY 11944, USA; 6 Departmenr of Libral Arts & Sciences, University of Illinois at Urbana-Champaign, Champaign, IL 61820, USA; vivian.odonnell@usda.gov

**Keywords:** African swine fever, ASFV, Ghana, genome

## Abstract

African swine fever (ASF) is a lethal disease of domestic pigs that has been causing outbreaks for over a century in Africa ever since its first discovery in 1921. Since 1957, there have been sporadic outbreaks outside of Africa; however, no outbreak has been as devastating and as far-reaching as the current pandemic that originated from a 2007 outbreak in the Republic of Georgia. Derivatives with a high degree of similarity to the progenitor strain, ASFV-Georgia/2007, have been sequenced from various countries in Europe and Asia. However, the current strains circulating in Africa are largely unknown, and 24 different genotypes have been implicated in different outbreaks. In this study, ASF isolates were collected from samples from swine suspected of dying from ASF on farms in Ghana in early 2022. While previous studies determined that the circulating strains in Ghana were p72 Genotype I, we demonstrate here that the strains circulating in 2022 were derivatives of the p72 Genotype II pandemic strain. Therefore, this study demonstrates for the first time the emergence of Genotype II ASFV in Ghana.

## 1. Introduction

African swine fever (ASF) is a deadly contagious hemorrhagic disease of domestic and wild pigs that was first reported in Kenya in 1921 [1]. ASF has continuously caused outbreaks throughout different parts of Africa since its discovery, with a few sporadic outbreaks outside of Africa that were resolved without spreading worldwide. In 2007, this situation began to change after an outbreak of Genotype II ASF occurred in the Republic of Georgia [2]. This outbreak continued to spread to neighboring countries, and in 2018 an outbreak occurred in China [3], followed by rapid spread of ASF throughout southeast Asia. In addition, the disease has continued to spread to east and central Europe. In 2021 an outbreak occurred in the Dominican Republic, marking the first outbreak in recent history of ASF in the western hemisphere [4].

The causative agent of the disease, ASF virus (ASFV), is a large DNA virus belonging to the family *Asfarviridae*; it contains a large dsDNA genome ranging from 170–192 kb that encodes 150 to 200 proteins [5,6,7,8,9]. Until recently the ability to perform full-length genome sequencing of the ASFV genome was costly and difficult, which has restricted full-length genome sequencing to only a very few virus isolates. Accordingly, ASFV strains are often broadly characterized by genotype; currently ASFV is classified by 24 genotypes based on the sequencing of a 478 bp fragment of the B646L gene, which encodes for the ASFV capsid protein p72 [10,11,12,13]. Further classifications have been implemented by combination of the sequences of genes encoding for p54, p72, and the central variable region (CVR) of pB602L [14]. However, due to the complexity inherent to the ASFV genome (large size, over 150 different proteins, different number of proteins between isolates, deletions and fusions within the multi-gene family (MGF) genes), classifications based on a small subset of genes are insufficient. Sequencing of the entire genome of ASFV can provide a better understanding of how many different ASFV genomes are currently circulating in the world, and perhaps allow for better prediction of cross-protection through sequence homology.

Currently, there is only one vaccine available for commercial use for ASF, and its use is limited to Vietnam, with several other experimental vaccines under different stages of development [15]. Therefore, in most of the world control of ASF relies on movement restrictions and culling of infected animals on farms. To more efficiently develop vaccines that can offer protection against different isolates, a more thorough classification of ASFV based on full length sequencing instead of p72 genotyping is needed. Indeed, the molecular basis of cross-protection between or within genotypes is largely unknown, and the significance of genotypes for predicting cross-protection is uncertain, as historically genotyping of ASFV was performed under convivence for disease tracking, rather than because any scientific reason suggested cross-protection within or between genotypes. However, it has been shown that live-attenuated ASFV with specific genetic deletions offers protection against the parental homologous strain of ASFV; see Gladue et.al., 2022 [15] for a review of ASFV vaccines.

Here, we sequenced the full genomes of ASFV circulating in 2022 in Ghana, revealing that they belong to Genotype II and are a derivative of the current pandemic strain ASFV-Georgia/2007. Full-genome sequencing of the 2022 outbreak strains in Ghana revealed a unique deletion in the 5′ end of the genome, completely deleting eight MGF genes. The remaining genes in the Ghana isolates of ASFV are a 99% match to the current pandemic strain. While previous reports determined that Genotype I was the main p72 genotype in Ghana causing outbreaks, this report determines for the first time that Genotype II of ASFV is causing outbreaks in domestic swine in Ghana [16]. The results presented here show the further spread of the pandemic strain of ASFV into Ghana and evidence genetic differences that would not be noted by purely genotyping these isolates by partial gene sequence analysis. This additional knowledge is important for future outbreak mapping to determine both the introduction of these isolates into new areas as well as to detect the introduction of new isolates into areas where outbreaks have previously occurred. This information is critical for both epidemiological control of the disease, and contributes to determining vaccine matching.

## 2. Materials and Methods

### 2.1. Sample Collection and Next Generation Sequencing

Four isolates of ASFV were obtained from swine from various regions of Ghana that were suspected to have died from ASF during the first few months of 2022. ASFV Ghana2002-35 was collected on 4 January 2022 from a spleen sample, ASFV isolate Ghana2022-34 was collected on 7 March 2022 from a kidney sample. ASFV isolate Ghana2022-40 was collected on 11 March 2022 from a spleen sample, and ASFV isolate Ghana 2022-62 was collected on 3 February 2022 from a spleen sample. ASFV isolates Ghana2022-34, Ghana2022-35, Ghana2022-40, and Ghana2022-62 were passed one time in blood-derived primary swine macrophage cultures produced as previously described in [17]. Viral genomes were sequenced using an Illumina Nextseq500 sequencing platform, as previously described [18]. Additionally, for assembly across low-complexity regions of the genome, Ghana2022-35 was sequenced using the Oxford nanopore minion [16] sequencing platform using previously described methods [19]. In brief, viral DNA was extracted from infected macrophage cultures using a nuclear extraction kit (Active Motif, Carlsbad, CA, USA). The cytoplasmic fraction was used for sequencing of ASFV DNA. Libraries were created using the Nextera XT kit (Illumnia, San Diego, CA, USA) following the manufacturer’s protocol. Sequence analysis was performed using CLC Genomics Workbench software (CLCBio, Waltham, MA, USA).

### 2.2. Genome Assembly

All steps were performed using the CLC Genomics Workbench (version 21). Illumina reads were trimmed for quality (limit = 0.05), ambiguous base pairs (max = 2), adapters, minimum size (min = 50), and from the 5′ (20 nucleotides) and 3′ terminal end (5 nucleotides). Ghana2022-35 was first constructed via de novo assembly using the following methodology. To remove reads resulting from the host sequence, 500,497,223 paired-end and 73,874 orphaned Illumina reads were mapped to ASFV strain ASFV-G (Accession: FR682468.2) and collected, resulting in 249,624 paired-end reads and 105,492 orphaned Illumina reads. Next, 131,918 Minion and the collected Illumina reads were entered into the “De Novo Assembly Long Reads and Polish with Short Reads” pipeline using the default parameters. From the assembly, a contig that matched to ASFV was extracted, and all Minion and trimmed Illumina reads were mapped back to the genome using the default parameters, resulting in an average depth of coverage of 313 reads. The consensus sequence was extracted, resulting in a 184,773 nt genome. To construct the genomes of Ghana2022-34, Ghana2022-40, and Ghana2022-62, Illumina reads originating from each sample were trimmed as previously described and separately mapped to the Ghana2022-35 genome, and the consensus sequences were extracted. Ambiguous nucleotides were manually resolved based on the mapped reads.

### 2.3. Annotation of the Genome

ORF prediction was performed on Ghana2022-35 using CLC Genomics workbench v21 using the “Find Open Reading Frames” module with the following parameters: both strands, min. size = 50 nt, genetic code = standard, start codon AUG, resulting in 427 ORFs. Protein translations of the ORFs were compared against all 195 protein sequences of the ASFV strain ASFV-G (Accession: FR682468.2) using the default parameters of BLASTP. ORFs with >80% sequence identity and coverage were annotated with the top ASFV-G protein hit. The strand and the start and end nucleotide positions for each gene were extracted from the output file and, when required, were manually edited to include the correct start and stop codon. To cull the remaining unannotated ORFs, the nucleotide sequences of the remaining ORFs were compared to the nucleotide sequence of ASFV-G using the default parameters of TBLASTN. ORFs that matched ASFV-G with over 80% identification and coverage were discarded as incorrectly predicted ORFs. Translated sequences from the remaining unannotated ORFs were compared to proteins translated from our previously described ASFV database [5]. ORFs with no matches or with matches to a hypothetical protein were removed, and the remaining ORFs were annotated with the top ASFV protein hit. The strand, start, and end nucleotide positions for each gene were extracted from the output file and manually edited when required to include the correct start and stop codon. Annotations were entered on the Ghana2022-35 genome in CLC Genomics Workbench, resulting in 167 annotations. Annotations were transferred to Ghana2022-34, Ghana2022-40, and Ghana2022-62 using the Genome Annotation Transfer Utility [20]. Concurrently, proteins with sequences that differed compared to Ghana2022-35 and annotations that failed to transfer were collected and recorded.

### 2.4. Identification of Single-Nucleotide Polymorphisms (SNPs)

Ghana2022-35 was mapped to ASFV-G (Accession: FR682468.2) using the Map Long Reads to Reference module in CLC Genomics with long-read splice alignment enabled. The Basic Variant Detection module within CLC Genomics was then used, followed by the Amino Acid Changes module. The same methodology was used with Ghana2022-34, Ghana2022-40, and Ghana2022-62 separately mapped to Ghana2022-35 in order to determine the SNPs between the samples originating from Ghana.

### 2.5. Genome Alignment

A database of 32 ASFV genomes was created by downloading the top 11 ASFV genomes on NCBI based on percent identity and coverage with respect to Ghana2022-34, Ghana2022-35, Ghana2022-40, and Ghana2022-62 using the default parameters of BLASTN [21,22,23,24]; the genomes of curated historical isolates were downloaded from 4virology.net accessed on, 21 May 2023 and any remaining full length ASFV sequences originating from Africa were downloaded from NCBI. Genomes were aligned against the sequences originating from Ghana using the Create Whole Genome Alignment module in CLC Genomics, and a phylogeny was constructed using four combinations of comparisons (Average Nucleotide Identity (ANI) or Average Alignment Percentage (AP)) and methods (Neighbor Joining (NJ) or Unweighted Pair Group Method with Arithmetic Mean (UPGMA)).

### 2.6. Protein Alignment

The protein translations from all annotated ORFs originating from the genomes isolated from Ghana were compared against the NCBI database using the default parameters of BLASTP [21,22,23,24]. Proteins containing unique sequences were collected along with their historical isolates’ homologs; these were downloaded from 4virology.net accessed on 21 May 2023 and aligned in CLC Genomics using the following parameters: Gap Open = 10, Gap extension = 1, End Gap Cost = free, Alignment = very accurate.

## 3. Results

### 3.1. Characteristics of Collected 2022 Outbreak Samples

**Figure 1:**
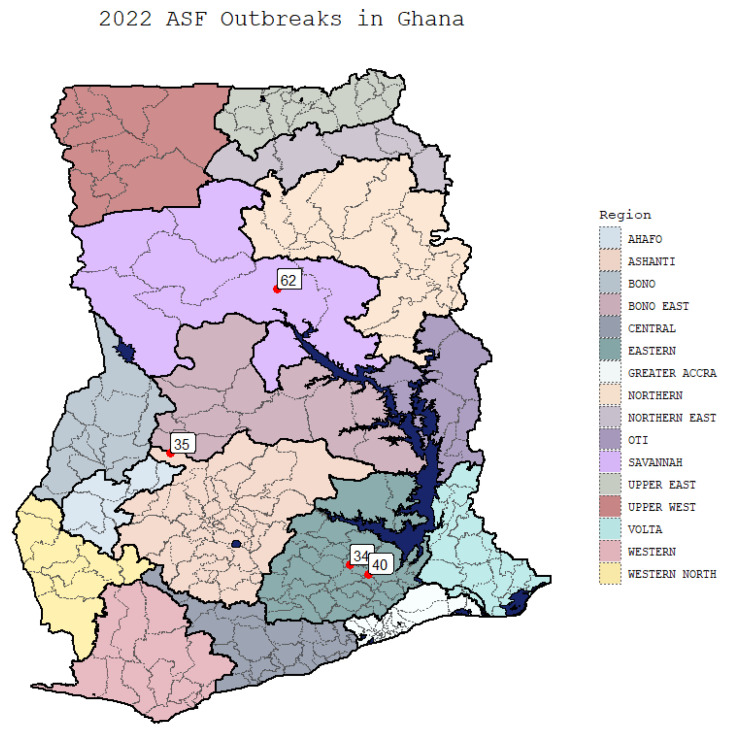
A map of the different regions in Ghana with the indicated outbreaks identified. Sample 35 from the Ashanti region was collected 4 January 2022; Sample 62 from the Northern region was collected 3 February 2022; Sample 34 was collected 7 March 2022; Sample 40 was collected 11 March 2022.

Four isolates of ASFV were obtained from outbreaks in the first few months 2022 from the Ashanti, Northern, and Eastern regions of Ghana (Figure 1). ASFV Ghana2002-35 was collected on 4 January 2022 from the Ashanti region during an outbreak at a small farm with 20 head of swine, where 11 swine had died and 8 additional swine were showing clinical signs of ASF including red patches on the skin, vomiting blood, swollen head, and were off feed. The ASFV isolate was sequenced from a spleen sample obtained from one of the animals that died. ASFV isolate Ghana2022-34 was obtained from the Eastern region of Ghana on 7 March 2022 from a farm with 416 head of swine, where 186 swine were dead and 45 additional animals were showing clinical signs of ASF, including abortion, loss of coordination, difficulty breathing, and were off feed. The virus was isolated from a kidney sample obtained from an animal that died. ASFV isolate Ghana2022-40 was obtained from the Eastern region of Ghana on 11 March 2022 from a farm containing 50 head of swine, where 4 had died and 11 more were showing clinical symptoms, including loss of appetite, dullness and weakness, reluctance to stand, red pigmentation of the skin, fever, and difficulty breathing. A spleen was collected from one of the animals that died, and the ASFV isolate was sequenced. ASFV isolate Ghana 2022-62 was obtained in the Northern region of Ghana from an outbreak occurring on February 3, 2022 at a farm consisting of 70 head of swine, with 59 swine found dead and 8 more showing clinical symptoms consisting of anorexia, lethargy, and hyperemia of the whole body. The virus was obtained from a spleen sample from one of the animals that died and was sequenced. After the clinical sample was submitted and confirmed positive, no further information was provided by the farmers about the outcome of the outbreak. Nonetheless, these samples from three distinct regions of Ghana (Figure 1) can provide an idea of the circulating strains of ASFV.

### 3.2. Genotyping of the ASFV Isolates

Genotyping was performed, and all four isolates (Ghana2022-34, Ghana2022-35, Ghana2022-40, and Ghana2022-62) were determined to be Genotype II from the coding sequences of p72 (B646L) and Genotype IIa based on p54 (E183L) (Table 1).

### 3.3. ASFV Full-Genome Alignments

**Figure 2:**
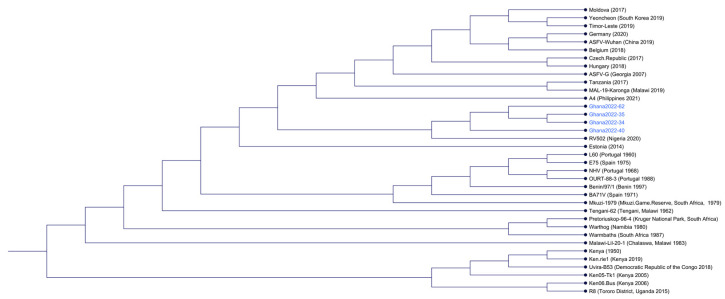
Whole genome alignment of ASFV isolates using API and NJ. Ghana genomes are highlighted in blue font.

Using whole-genome alignments for ASFV isolates available on NCBI, phyologenic trees were constructed using four different parameters: ANI + NJ, AP +NJ, UPGMA + ANI, and UPGMA + NJ. All methods grouped the Ghana 2022 genomes (blue font) together as a sister group to RV502 (Accession: OP672342), a 2020 isolate from Nigeria [25]. However further analysis relied only on ANI + NJ, as it correctly grouped the prototypical isolates. The Ghana 2022 genomes shared a sister group with Estonia-2014 (Accession: LS478113.1) as well (Figure 2). RV502 was recently shown to have a ~6500 bp deletion resulting in the loss of MGF_110-8L, MGF_110-XR, ACD 00190, MGF_110-9L, ACD 00210, MGF_110-10L-14 L, G ACD 00240, MGF_110-12L, MGF_110-13La, MGF_110-13Lb, ACD 00270, MGF_360-4L, ACD 00300, and G ACD 00350 [25]. Estonia-2014 has been previously shown to have a ~14,500 bp deletion of the 5′ end, resulting in the loss of the MGF genes MGF_110-1L through MGF_110-14L and MGF_360-1L through MGF_360-3L, along with the partial deletion of MGF_110-13 L [26].

### 3.4. Genetic Variation between Ghana 2022 Isolates

To determine genetic differences between the four Ghana isolates, the genomes of Ghana2022-34, Ghana2022-40, and Ghana2022-62 were compared to the first outbreak strain in Ghana identified in 2022, Ghana2022-35 (Supplementary Table S1). Note that the following genes could not be compared because of gaps in the assembly: EP402R (Ghana2022-34, Ghana2022-40, and Ghana2022-62), DP79L (Ghana2022-62), EP153R (Ghana2022-34 and Ghana2022-40), and MGF_360-6L (Ghana2022-40).

Comparison between the Ghana2022-35 and Ghana2022-34 genome resulted in 34 SNPs. Comparison of the genome of Ghana2022-35 and Ghana2022-40 resulted in 26 SNPs, seven of which led to a change in the amino acid (AA) sequence of seven ORFs: X69R (P16L), MGF_505-1R (R450I), K145R (Y166H), C315R (Q30H), E199L (P85A), MGF_505-11L (I280V), and a frameshift in MGF_360-13L at A262. Comparison of the genome Ghana2022-35 and Ghana2022-62 contained thirteen SNPs, twelve of which led to a change in AA sequence of six ORFs: X69R (P16L), C475L (Q148H), E199L (P85A), MGF_505-11L (I280V), a frameshift in MGF_360-13L at A262 and a frameshift in MGF_360-18R at L54.

These results show that even in early 2022 there were unique genomic features at both the nucleotide and protein level between closely related outbreaks of ASFV in Ghana, suggesting variation between isolates causing outbreaks even within a restricted area and time of outbreak in Ghana.

### 3.5. Analysis of a 6534 nt Deletion and Individual MGFs

**Figure 3:**
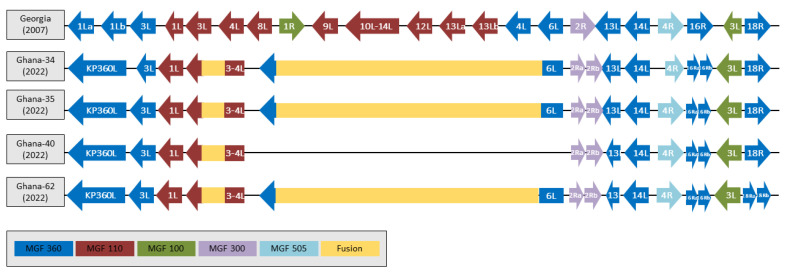
Graphic illustrating MGF genes that contain truncations, elongations, and fusions encoded by the 2022 Ghana isolates compared to the original outbreak strain ASFV-Georgia/2007. Note that the MGF_360-6L fusion was not annotated in Ghana2022-34 because of a single nucleotide gap in the genome.

When Ghana2022-35 was compared to the original outbreak strain ASFV-Georgia/2007, there were a total of 69 SNP, insertions, and deletions, of which 37 resulted in an AA change in 34 ORFs, as detailed in Supplementary Table S2. Compared to ASFV-Georgia/2007, there was a 6534 nt deletion in the Ghana genomes that is not present in other isolates that are decedents of the original Georgia/2007 outbreak. This deletion is between the coding regions of MGF_110-8L and MGF_360-6L, and resulted in the loss of the multi-gene family (MGF) proteins MGF_110-8L, MGF_100-1R, MGF_110-9L, 0 MGF_110-10-14L fusion protein, MGF_110-12L, MGF_110-13La, MGF_110-13Lb, and MGF_360-4L. This deletion additionally consists of the open reading frames (ORFs) ASFV G ACD 00190, ASFV G ACD 00300, ASFV G ACD 00210, ASFV G ACD 00240, and ASFV G ACD 00270. This deletion resulted in the replacement of the 35 C-terminal of MGF_360-6L with the sequence “RFTTNPLSS*” that originated from an off-frame translation within the original MGF_110-8L ORF (Figure 3). Further, a mutation led to the loss of the start methionine codon in the genome that originated from Ghana2022-40, leading to a further shortening of MGF_360-6L. When compared to ASFV-G, an additional 688 nt deletion was observed within the Ghana isolates, which created an MGF_110-3L-4L fusion protein (Figure 3). Further differences that led to truncation or extensions were seen in the coding sequences of other MGF proteins (Supplementary Table S2 and Table 2). KP360L is a fusion of MGF_360-1la and MGF_360-1lb resulting from a single nucleotide insertion in MGF_360-1Lb (Figure 3 and Supplementary Figure S1), though the amino acid sequence is closer to ASFV-G MGF_360-1La and MGF_360-1Lb than to the sequence of KP360L encoded by historical ASFV isolates. Additional differences in the genes of the MGF families include an extension of MGF_110-1L (all Ghana 2022 genomes), a truncation of MGF_360-13L (all 2022 Ghana genomes, and samples Ghana2022-40 and Ghana2022-62 are further truncated compared to Ghana2022-34 and 35), a truncation of MGF_360-16R that may possibly lead to the encoding of MGF_360-16Ra and MGF_360-16Rb (all 2022 Ghana genomes), a truncation of MGF_300-2R that may possibly lead to the encoding of MGF_300-2Ra and MGF_300-2Rb (all Ghana genomes), an extension of MGF_100-3L (all Ghana 2022 genomes), and a truncation of MGF_360-18R that may lead to the encoding of MGF_360-18Ra and MGF_360-18Rb (Ghana2022-62 only). Mutations that led to the encoding of MGF genes with unique AA sequences are further explored in the next section.

### 3.6. Analysis of Ghana 2022 Proteins

All proteins encoded by Ghana2022-34, Ghana2022-35, Ghana2022-40, and Ghana2022-62 were submitted to BLASTP on NCBI [21,22,23,24]. Sequences that did not have 100% identity and 100% coverage matches were considered to be unique protein sequences specific to the 2022 Ghana genomes. As summarized in Table 2, the following proteins encoded from the specified Ghana genome contained unique amino acid sequences that can be used as markers for the 2022 Ghana genomes (Supplementary Figure S1): B117L (Y98F, all genomes), B602L (V13I, all genomes), C315R (Q30H, Ghana2022-40), C475L (Q148H, Ghana2022-62), EP402R (L300V and P313L, Ghana2022-35), EP424R (N155S, all genomes), F334L (S282G, all genomes), H339R (Q319R, all genomes), K145R (Y116R, Ghana2022-40), KP360L (F19L, H41Y, and K47E, all genomes), MGF_110-1L (an additional 54 amino acids to the C-terminus, all genomes), MGF_110-7L (Y112, all genomes), MGF_360-13L (G177D, all genomes, NINQAM-LTSVQYYNIGNIFFCID 262-284 ATSTKLCLLQYNIITSVIYFSV*, (Ghana2022-34 and Ghana2022-35 and NINQAMLTSVQYYN 262-276 QHQPSYAY-FSTIL*, (Ghana2022-40 and Ghana2022-62), MGF_360-14L (P351L, all genomes), MGF_360-16R (split into MGF_360-16Ra and MGF_360-16Rb, all genomes), MGF_360-18R (split into MGF_360-168a and MGF_360-18Rb, Ghana2022-62), MGF_360-6L (LHKKILEPSE 341-350 RFTTNPLSS*, Ghana2022-34, Ghana2022-35, and Ghana2022-62), MGF_505-11L (S231L, all genomes) and (I280V, Ghana2022-40 and Ghana2022-62), MGF_505-1R (I450R, all genomes), MGF_505-2R (T355A, all genomes), MGF_505-5R (G477S, all genomes), NP868R (A9T and A589V, all genomes), and X69R (L16P, Ghana2022-34 and Ghana2022-35).

## 4. Discussion

Here, we report four full-length sequences from the 2022 outbreaks in Ghana. This is the first report of Genotype II strains circulating in Ghana that are derivatives of the current Eurasia strain that caused the 2007 outbreak in the Republic of Georgia, as previous outbreaks of ASFV in Ghana were identified as Genotype I [16]. It is interesting that derivatives of ASF can occur with distinct genetic markers even within a few months of outbreaks. However, common markers can be seen when evaluating the differences between the four isolates, suggesting that they originated from the same parental strain or outbreak in recent history.

While the exact number of ASFV strains currently circulating in Africa is unknown, the information provided by full-genome sequencing of outbreak strains is imperative to track the continued evolution of ASFV strains. Historically, genotyping was performed to track ASF outbreaks out of convenience; however, partial genome sequencing does not provide an accurate representation of the currently circulating strains. This study and others [25,26,27] based on ASFV whole-genome sequencing clearly indicate that there are different circulating strains of Genotype II, an observation that would be missed by only sequencing a few selected genes. For example, the isolates from Ghana have a deletion of eight MGF family genes. Recently, a 6534 nt deletion, MGF_110-3L-4L fusion, and MGF_360-1La-ILb fusion were observed in RV502 [25]. Interestingly, unlike RV502, the Ghana isolates do not contain the reverse complement duplication of the 5′ end of the genome on the 3′ end of the genome, and no fusion between MGF_360-21L and MGF_360-2L was observed [25]. There have been previous reports that deletion of six MGF genes can result in full or partial attenuation of ASFV [28,29,30,31,32,33]. It is important to note that the deletion observed in the Ghana isolates is not the same or even close to the deletion observed in the genetically engineered strains that were attenuated. Other isolates such as Nigeria-RV502 had deletions of MGF genes; however, these do not show any decreased virulence in domestic swine [25]. Conversely, large deletions that include many MGF genes of the p72 Genotype I or Genotype II strains have been found in animals surviving outbreaks [25,34], although when tested in swine these strains had a level of residual virulence. It should be noted that there are multiple families of MGF genes. Furthermore, even MGF genes within the same family appear to be unique based on structural prediction [9], suggesting that different MGF genes have different specific molecular functions and that different deletions of specific MGF genes would have a differential effect on ASFV [35]. It is possible that these genetic deletions have occurred as a fit for a purposely smaller genome in domestic swine, and that these strains could have reduced replication in ticks (as was observed in other isolates [36]) or perhaps in wild suidae; however, further experiments would have to be performed to evaluate this theory.

Although in this study there were minor differences in ASFV proteins when comparing the genomes of the outbreak strains in Ghana to that of the original ASFV-Georgia/2007 backbone, our results would nonetheless suggest that vaccines using the ASFV-Georgia/2010 backbone [28,37,38,39,40] would be effective against the outbreak strain occurring in Ghana, as previous reports using the ASFV-G-ΔI177L vaccine strain have determined this vaccine to be effective against both the original ASFV-Georgia/2010 strain and recent strains in Vietnam [41]. It is important to note that domestic pigs in Ghana are a crossbreed between European pigs and the local Ashanti Dwarf pig, a breed that is noted for being hardy and less susceptible to local diseases [42]. It would be expected that an ASFV vaccine would show similar results in these pigs, as it did in local Vietnamese breeds [41], though the efficacy of any vaccine would have to be tested under local conditions. When vaccination becomes a method of controlling ASF worldwide, it will become increasingly important to determine the full-length genome of ASFV in order to accurately predict the effectiveness of vaccines rather than relying on one individual protein, as historically used out of convenience for genotyping, particularly as the persistence of Genotype II continues to evolve throughout different parts of the world. The information presented in this manuscript is important, as we have discovered currently circulating strains of ASFV in Africa, providing information on the potential to use either commercial or experimental ASF vaccines in particular areas.

## Figures and Tables

**Table 1:** Origin and genotyping of Ghana samples.

Sample	GenBank Accession	Location of Animal/Farm	Region	Date of Outbreak	Genotype p72/VP73 (B646L)	Genotype p54 (E183L)
35	OP479889	AKUMADAN	Ashanti	1/4/2022	II	IIa
62	OP718535	CENTRAL GONJA DISTRICT	Northern	2/3/2022	II	IIa
34	OP718533	BUNSO	Eastern	3/7/2022	II	IIa
40	OP718534	SUHYEN	Eastern	3/11/2022	II	IIa

**Table 2:** Proteins with unique amino acid sequences encoded by the indicated Ghana samples.

Gene	Ghana2022-34	Ghana2022-35	Ghana2022-40	Ghana2022-62
B117L	Y98F	Y98F	Y98F	Y98F
B602L	V13I	V13I	V13I	V13I
C315R	no mutation	no mutation	Q30H	no mutation
C475L	no mutation	no mutation	no mutation	Q148H
EP402R	na	L300V, P313L	na	na
EP424R	N155S	N155S	N155S	N155S
F334L	S282G	S282G	S282G	S282G
H339R	Q319R	Q319R	Q319R	Q319R
K145R	no mutation	no mutation	Y116R	no mutation
KP360L	F19L, H41Y, K47E	F19L, H41Y, K47E	F19L, H41Y, K47E	F19L, H41Y, K47E
MGF_110-1L	54 AA extension to the C-terminus	54 AA extension to the C-terminus	54 AA extension to the C-terminus	54 AA extension to the C-terminus
MGF_110-7L	Y112R	Y112R	Y112R	Y112R
MGF_360-13L	G177D, NINQAMLTSVQYYNIGNIFFCID 262-284 ATSTKLCLLQYNIITSVIYFSV*	G177D, NINQAMLTSVQYYNIGNIFFCID 262-284 ATSTKLCLLQYNIITSVIYFSV*	G177D, NINQAMLTSVQYYN 262-276 QHQPSYAYFSTIL*	G177D, NINQAMLTSVQYYN 262-276 QHQPSYAYFSTIL*
MGF_360-14L	P351L	P351L	P351L	P351L
MGF_360-16R	D92*, split into MGF_360-16Ra and MGF_360-16Rb	D92*, split into MGF_360-16Ra and MGF_360-16Rb	D92*, split into MGF_360-16Ra and MGF_360-16Rb	D92*, split into MGF_360-16Ra and MGF_360-16Rb
MGF_360-18R	no mutation	no mutation	no mutation	Split into MGF 360-18Ra and MGF 360-18Rb
MGF_360-6L	LHKKILEPSE 341-350 RFTTNPLSS*	LHKKILEPSE 341-350 RFTTNPLSS*	na	LHKKILEPSE 341-350 RFTTNPLSS*
MGF_505-11L	S231L	S231L	S231L, I280V	S231L, I280V
MGF_505-1R	I450R	I450R	I450R	I450R
MGF_505-2R	T355A	T355A	T355A	T355A
MGF_505-5R	G477S	G477S	G477S	G477S
NP868R	A9T, A589V	A9T, A589V	A9T, A589V	A9T, A589V
X69R	L16P	L16P	no mutation	no mutation

na: Gene was not annotated.

## Data Availability

Genome sequences have been deposited in GenBank under the accession nos. OP718533 (Ghana2022-34), OP479889 (Ghana2022-35), OP718534 (Ghana2022-40), and OP718535 (Ghana2022-62). Raw data for this project can be found in the GenBank SRA under accession nos. SRR22030039 to SRR22030091 in BioProject accession no. PRJNA894113.

## Supplementary Materials

The following supporting information can be downloaded at: https://www.mdpi.com/article/10.3390/v15081722/s1, Figure S1: Protein Alignments; Table S1: SNPs present in Ghana2022-34, Ghana2022-40 and Ghana2022-62 compared to Ghana2022-35; Table S2: SNPs present in Ghana2022-45 compared to ASFV-G.
