# Supplementary material for: The 2022 Outbreaks of African Swine Fever Virus Demonstrate the First Report of Genotype II in Ghana
Source: Viruses. 2023 Aug 11;15(8):1722. doi: 10.3390/v15081722 (PMC10459280; doi:10.3390/v15081722)
Supplement: Supplementary file 1 [file viruses-15-01722-s001.zip › Supplementary Figure S1- Protein Alignments (1).pdf]

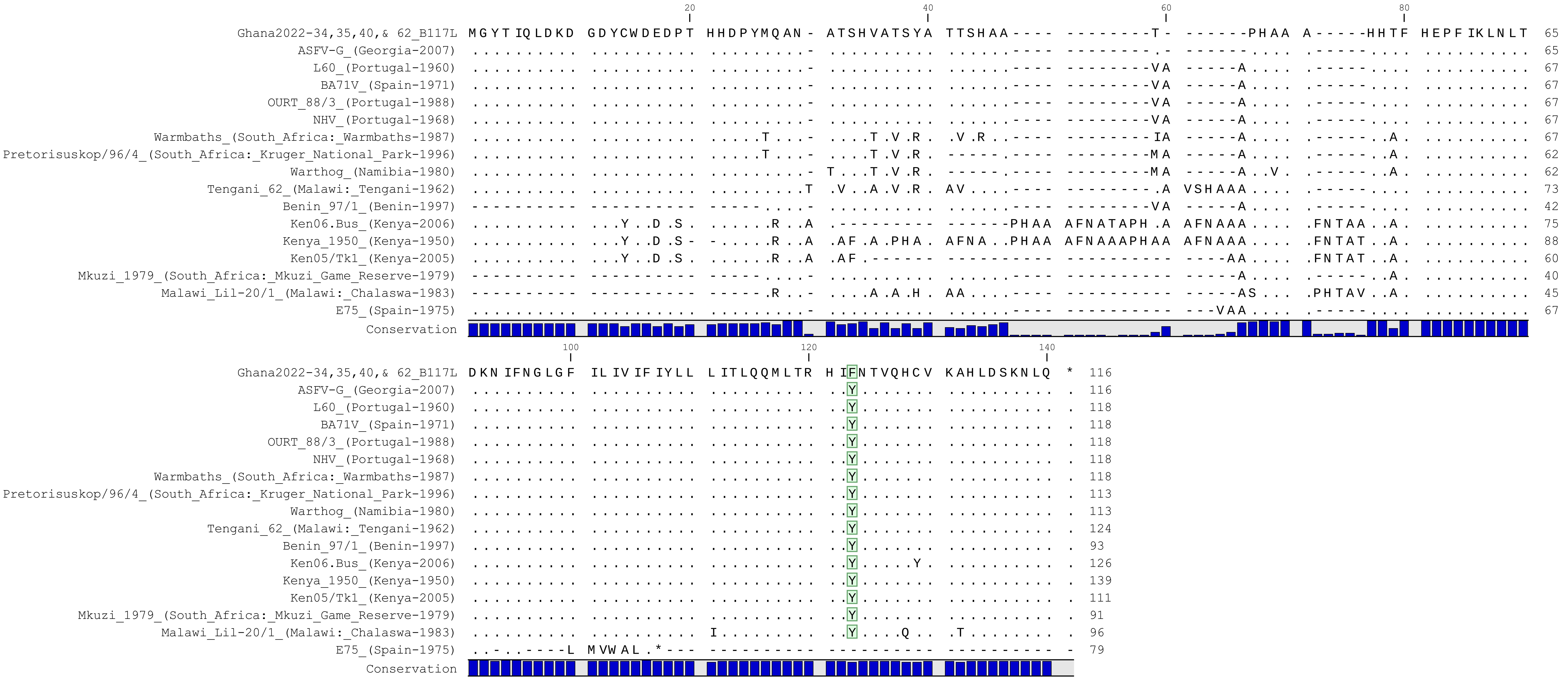
20
40
60
80
Ghana2022-34,35,40,& 62_B117L MGYTIQLDKD GDYCWDEDPT HHDPYMQAN- ATSHVATSYA TTSHAA---- --------T- ------PHAA A----HHTF HEPFIKLNLT 65
ASFV-G_(Georgia-2007) 65
L60_(Portugal-1960) 67
BA71V_(Spain-1971) 67
OURT_88/3_(Portugal-1988) 67
NHV_(Portugal-1968) 67
Warmbaths_(South_Africa:_Warmbaths-1987) 67
Pretorisuskop/96/4_(South_Africa:_Kruger_National_Park-1996) 62
Warthog_(Namibia-1980) 62
Tengani_62_(Malawi:_Tengani-1962) 73
Benin_97/1_(Benin-1997) 42
Ken06.Bus_(Kenya-2006) 75
Kenya_1950_(Kenya-1950) 88
Ken05/Tk1_(Kenya-2005) 60
Mkuzi_1979_(South_Africa:_Mkuzi_Game_Reserve-1979) 40
Malawi_Lil-20/1_(Malawi:_Chalaswa-1983) 45
E75_(Spain-1975) 67
Conservation
100
120
140
Ghana2022-34,35,40,& 62_B117L DKNIFNGLGF ILIVIFIYLL LITLQQMLTR HIFNTVQHCV KAHLDSKNLQ * 116
ASFV-G_(Georgia-2007) 116
L60_(Portugal-1960) 118
BA71V_(Spain-1971) 118
OURT_88/3_(Portugal-1988) 118
NHV_(Portugal-1968) 118
Warmbaths_(South_Africa:_Warmbaths-1987) 118
Pretorisuskop/96/4_(South_Africa:_Kruger_National_Park-1996) 113
Warthog_(Namibia-1980) 113
Tengani_62_(Malawi:_Tengani-1962) 124
Benin_97/1_(Benin-1997) 93
Ken06.Bus_(Kenya-2006) 126
Kenya_1950_(Kenya-1950) 139
Ken05/Tk1_(Kenya-2005) 111
Mkuzi_1979_(South_Africa:_Mkuzi_Game_Reserve-1979) 91
Malawi_Lil-20/1_(Malawi:_Chalaswa-1983) 96
E75_(Spain-1975) 79
Conservation

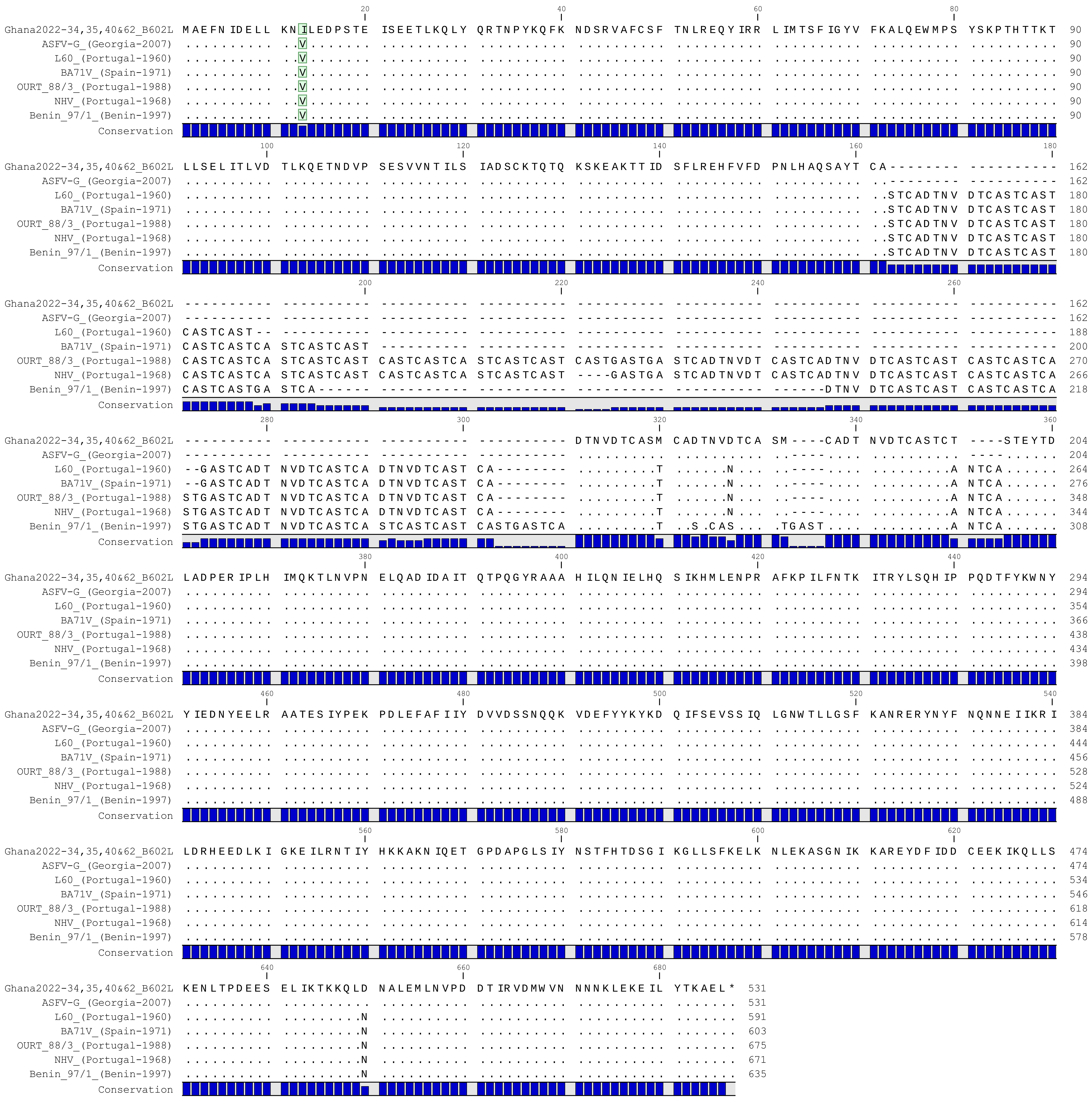

20 40 60 80
Ghana2022-34,35,40&62_B602L MAEFNIDELL KNILEDPSTE ISEETLKQLY QRTNPYKQFK NDSRVAFCSF TNLREQYIRR LIMTSFIGYV FKALQEWMPS YSKPTHTTKT 90
ASFV-G_(Georgia-2007) ....V....... 90
L60_(Portugal-1960) ....V....... 90
BA71V_(Spain-1971) ....V....... 90
OURT_88/3_(Portugal-1988) ....V....... 90
NHV_(Portugal-1968) ....V....... 90
Benin_97/1_(Benin-1997) ....V....... 90
Conservation
100 120 140 160 180
Ghana2022-34,35,40&62_B602L LLSELITLVD TLKQETNDVP SESVVNTILS IADSCKTQTQ KSKEAKTTID SFLREHFVFD PNLHAQSAYT CA-------- ---------- 162
ASFV-G_(Georgia-2007) 162
L60_(Portugal-1960) ....STCADTNV DTCASTCAST 180
BA71V_(Spain-1971) ....STCADTNV DTCASTCAST 180
OURT_88/3_(Portugal-1988) ....STCADTNV DTCASTCAST 180
NHV_(Portugal-1968) ....STCADTNV DTCASTCAST 180
Benin_97/1_(Benin-1997) ....STCADTNV DTCASTCAST 180
Conservation
200 220 240 260
Ghana2022-34,35,40&62_B602L ---------- ---------- ---------- ---------- ---------- ---------- ---------- ---------- ---------- 162
ASFV-G_(Georgia-2007) ---------- ---------- ---------- ---------- ---------- ---------- ---------- ---------- ---------- 162
L60_(Portugal-1960) CASTCAST-- ---------- ---------- ---------- ---------- ---------- ---------- ---------- ---------- 188
BA71V_(Spain-1971) CASTCASTCA STCASTCAST ---------- ---------- ---------- ---------- ---------- ---------- ---------- 200
OURT_88/3_(Portugal-1988) CASTCASTCA STCASTCAST CASTCASTCA STCASTCAST CASTGASTGA STCADTNVDT CASTCADTNV DTCASTCAST CASTCASTCA 270
NHV_(Portugal-1968) CASTCASTCA STCASTCAST CASTCASTCA STCASTCAST ----GASTGA STCADTNVDT CASTCADTNV DTCASTCAST CASTCASTCA 266
Benin_97/1_(Benin-1997) CASTCASTGA STCA------ ---------- ---------- ---------- ---------- ------DTNV DTCASTCAST CASTCASTCA 218
Conservation
280 300 320 340 360
Ghana2022-34,35,40&62_B602L ---------- ---------- ---------- ---------- DTNVDTCASM CADTNVDTCA SM----CADT NVDTCASTCT ----STEYTD 204
ASFV-G_(Georgia-2007) ---------- ---------- ---------- ---------- ....----.... .... ----...... 204
L60_(Portugal-1960) --GASTCADT NVDTCASTCA DTNVDTCAST CA-------- ....T ......N... ..----.... ....A NTCA...... 264
BA71V_(Spain-1971) --GASTCADT NVDTCASTCA DTNVDTCAST CA-------- ....T ......N... ..----.... ....A NTCA...... 276
OURT_88/3_(Portugal-1988) STGASTCADT NVDTCASTCA DTNVDTCAST CA-------- ....T ......N... ..----.... ....A NTCA...... 348
NHV_(Portugal-1968) STGASTCADT NVDTCASTCA DTNVDTCAST CA-------- ....T ......N... ..----.... ....A NTCA...... 344
Benin_97/1_(Benin-1997) STGASTCADT NVDTCASTCA STCASTCAST CASTGASTCA ....T ..S.CAS... .TGAST.... ....A NTCA...... 308
Conservation
380 400 420 440
Ghana2022-34,35,40&62_B602L LADPERIPLH IMQKTLNVPN ELQADIDAIT QTPQGYRAAA HILQNIELHQ SIKHMLENPR AFKPILFNTK ITRYLSQHIP PQDTFYKWNY 294
ASFV-G_(Georgia-2007) 294
L60_(Portugal-1960) 354
BA71V_(Spain-1971) 366
OURT_88/3_(Portugal-1988) 438
NHV_(Portugal-1968) 434
Benin_97/1_(Benin-1997) 398
Conservation
460 480 500 520 540
Ghana2022-34,35,40&62_B602L YIEDNYEELR AATESIYPEK PDLEFAFIIY DVVDSSNQQK VDEFYYKYKD QIFSEVSSIQ LGNWTLLGSF KANRERYNYF NQNNEIIKRI 384
ASFV-G_(Georgia-2007) 384
L60_(Portugal-1960) 444
BA71V_(Spain-1971) 456
OURT_88/3_(Portugal-1988) 528
NHV_(Portugal-1968) 524
Benin_97/1_(Benin-1997) 488
Conservation
560 580 600 620
Ghana2022-34,35,40&62_B602L LDRHEEDLKI GKEILRNTIY HKKAKNIQET GPDAPGLSIY NSTFHTDSGI KGLLSFKELK NLEKASGNIK KAREYDFIDD CEEKIKQLLS 474
ASFV-G_(Georgia-2007) 474
L60_(Portugal-1960) 534
BA71V_(Spain-1971) 546
OURT_88/3_(Portugal-1988) 618
NHV_(Portugal-1968) 614
Benin_97/1_(Benin-1997) 578
Conservation
640 660 680
Ghana2022-34,35,40&62_B602L KENLTPDEES ELIKTKKQLD NALEMLNVPD DTIRVDMWVN NNNKLEKEIL YTKAEL* 531
ASFV-G_(Georgia-2007) 531
L60_(Portugal-1960) ....N 591
BA71V_(Spain-1971) ....N 603
OURT_88/3_(Portugal-1988) ....N 675
NHV_(Portugal-1968) ....N 671
Benin_97/1_(Benin-1997) ....N 635
Conservation

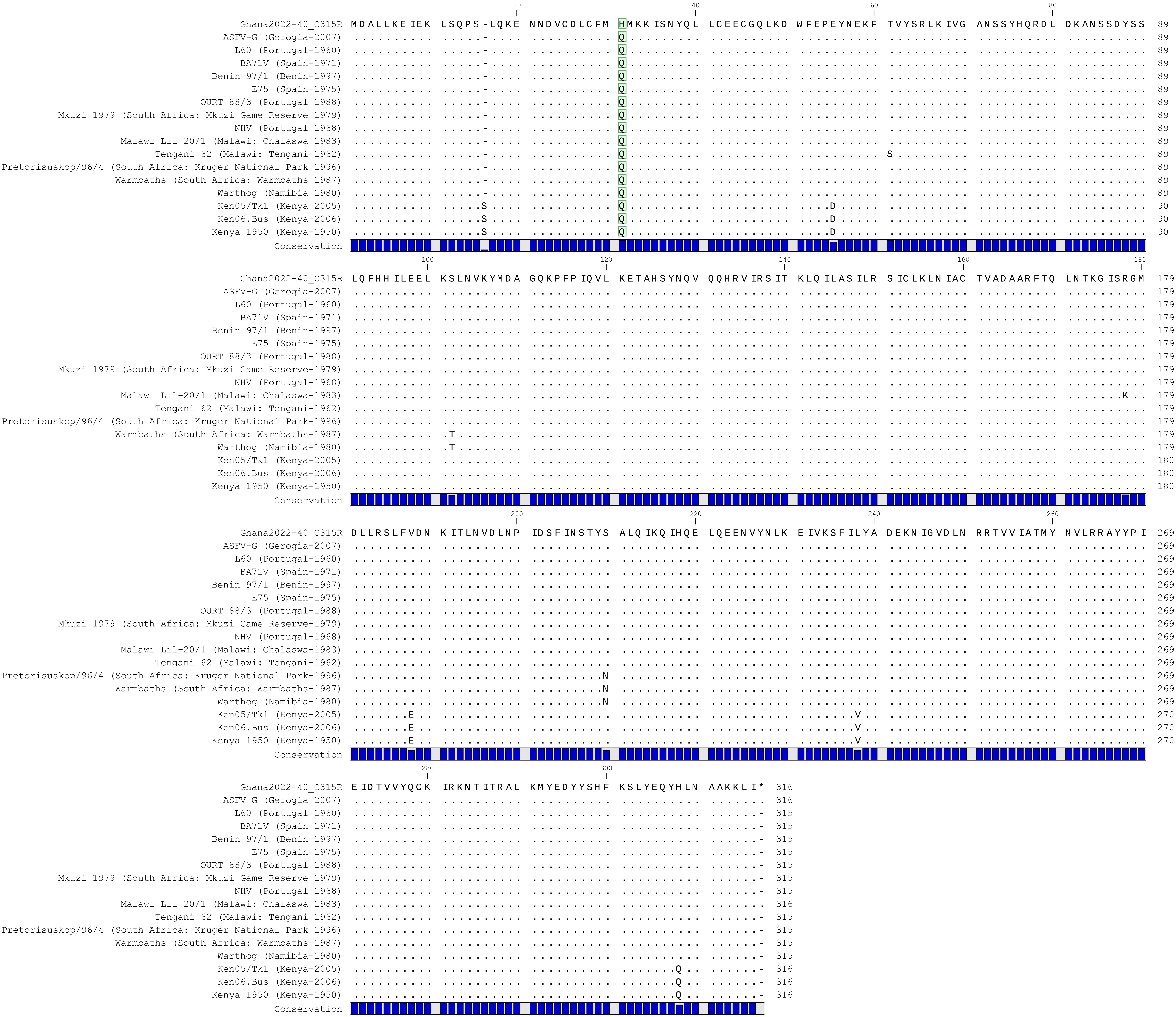

Ghana2022-40_C315R
ASFV-G (Gerogia-2007)
L60 (Portugal-1960)
BA71V (Spain-1971)
Benin 97/1 (Benin-1997)
E75 (Spain-1975)
OURT 88/3 (Portugal-1988)
Mkuzi 1979 (South Africa: Mkuzi Game Reserve-1979)
NHV (Portugal-1968)
Malawi Lil-20/1 (Malawi: Chalaswa-1983)
Tengani 62 (Malawi: Tengani-1962)
Pretorisuskop/96/4 (South Africa: Kruger National Park-1996)
Warmbaths (South Africa: Warmbaths-1987)
Warthog (Namibia-1980)
Ken05/Tk1 (Kenya-2005)
Ken06.Bus (Kenya-2006)
Kenya 1950 (Kenya-1950)
Conservation
MDALLKEIEK LSQPS-LQKE NNDVCDLCFM HMKKISNYQL LCEECGQLKD WFEPEYNEKF TVYSRLKIVG ANSSYHQRDL DKANSSDYSS 89
LQFHHILEEL KSLNVKYMDA GQKPFPIQVL KETAHSYNQV QQHRVIRSIT KLQILASILR SICLKLNIAC TVADAARFTQ LNTKGISRGM 179
DLLRSLFVDN KITLNVDLNP IDSFINSTYS ALQIKQIHQE LQEENVYNLK EIVKSFILYA DEKNIGVDLN RRTVVIATMY NVLRRAYYPI 269
EIDTVVYQCK IRKNTITRAL KMYEDYYSHF KSLYEQYHLN AAKKLI* 316

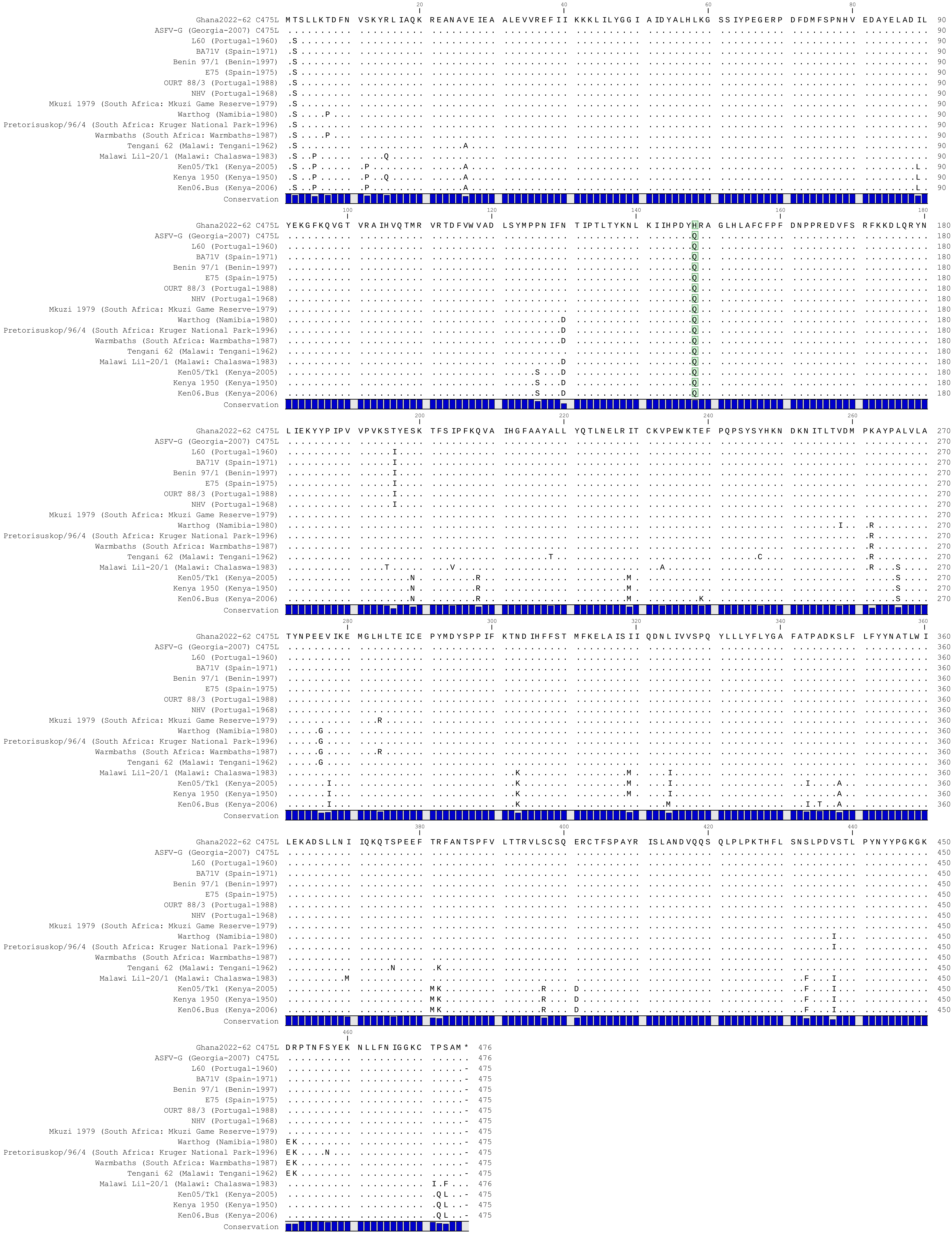

Ghana2022-62 C475L
ASFV-G (Georgia-2007) C475L
L60 (Portugal-1960)
BA71V (Spain-1971)
Benin 97/1 (Benin-1997)
E75 (Spain-1975)
OURT 88/3 (Portugal-1988)
NHV (Portugal-1968)
Mkuzi 1979 (South Africa: Mkuzi Game Reserve-1979)
Warthog (Namibia-1980)
Pretorisuskop/96/4 (South Africa: Kruger National Park-1996)
Warmbaths (South Africa: Warmbaths-1987)
Tengani 62 (Malawi: Tengani-1962)
Malawi Lil-20/1 (Malawi: Chalaswa-1983)
Ken05/Tk1 (Kenya-2005)
Kenya 1950 (Kenya-1950)
Ken06.Bus (Kenya-2006)
Conservation
MTSLLKTDFN VSKYRLIAQK REANAVEIEA ALEVVREFII KKKLILYGGI AIDYALHLKG SSIYPEGERP DFDMFSPNHV EDAYELADIL 90
YEKGFKQVGT VRAIHVQTMR VRTDFVWVAD LSYMPPNIFN TIPTLTYKNL KIIHPDYHRA GLHLAFCFPF DNPPREDVFS RFKKDLQRYN 180
LIEKYYPIPV VPVKSTYESK TFSIPFKQVA IHGFAAYALL YQTLNELRIT CKVPEWKTEF PQPSYSYHKN DKNITLTVDM PKAYPALVLA 270
TYNPEEVIKE MGLHLTEICE PYMDYSPPIF KTNDIHFFST MFKELAISII QDNLIVVSPQ YLLLYFLYGA FATPADKSLF LFYYNATLWI 360
LEKADSLLNI IQKQTSPEEF TRFANTSPFV LTTRVLSCSQ ERCTFSPAYR ISLANDVQQS QLPLPKTHFL SNSLPDVSTL PYNYYPGKGK 450
DRPTNFSYEK NLLFNIGGKC TPSAM* 476

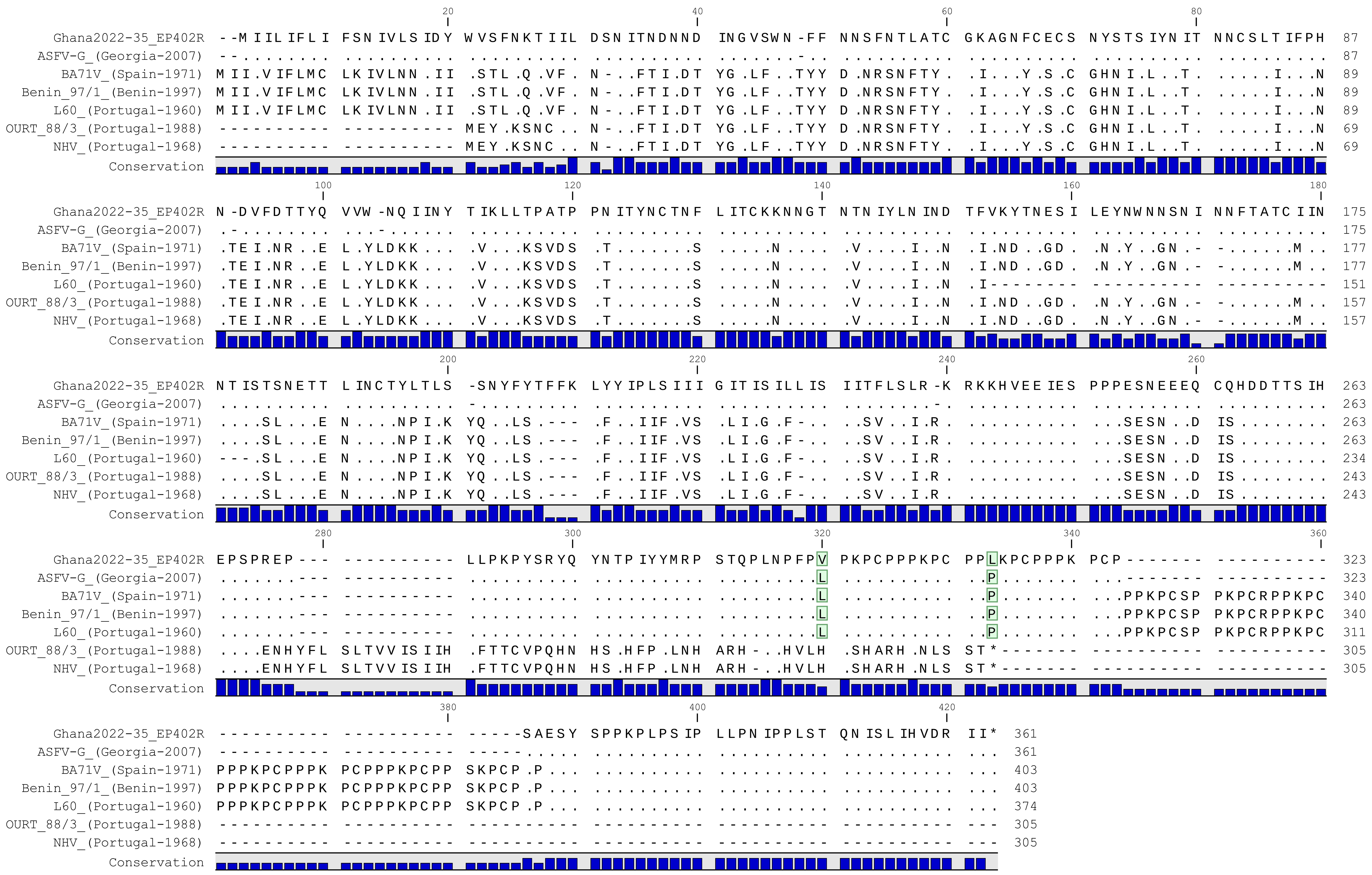

Ghana2022-35_EP402R --MIILIFLI FSNIVLSIDY WVSFNKTIIL DSNITNDNND INGVSWN-FF NNSFNTLATC GKAGNFCECS NYSTSIYNIT NNCSLTIFPH 87
ASFV-G_(Georgia-2007) --........ ....-.. 87
BA71V_(Spain-1971) MII.VIFLMC LKIVLNN.II .STL.Q.VF. N-..FTI.DT YG.LF..TYY D.NRSNFTY. .I...Y.S.C GHNI.L..T. .....I...N 89
Benin_97/1_(Benin-1997) MII.VIFLMC LKIVLNN.II .STL.Q.VF. N-..FTI.DT YG.LF..TYY D.NRSNFTY. .I...Y.S.C GHNI.L..T. .....I...N 89
L60_(Portugal-1960) MII.VIFLMC LKIVLNN.II .STL.Q.VF. N-..FTI.DT YG.LF..TYY D.NRSNFTY. .I...Y.S.C GHNI.L..T. .....I...N 89
OURT_88/3_(Portugal-1988) ---------- ---------- MEY.KSNC.. N-..FTI.DT YG.LF..TYY D.NRSNFTY. .I...Y.S.C GHNI.L..T. .....I...N 69
NHV_(Portugal-1968) ---------- ---------- MEY.KSNC.. N-..FTI.DT YG.LF..TYY D.NRSNFTY. .I...Y.S.C GHNI.L..T. .....I...N 69
Conservation
Ghana2022-35_EP402R N-DVFDTTYQ VVW-NQIINY TIKLLTPATP PNITYNCTNF LITCKKNNGT NTNIYLNIND TFVKYTNESI LEYNWNNSNI NNFTATCIIN 175
ASFV-G_(Georgia-2007) .-........ ...-...... 175
BA71V_(Spain-1971) .TEI.NR..E L.YLDKK... .V...KSVDS .T.......S ....N..... .V....I..N .I.ND..GD. .N.Y..GN.- -......M.. 177
Benin_97/1_(Benin-1997) .TEI.NR..E L.YLDKK... .V...KSVDS .T.......S ....N..... .V....I..N .I.ND..GD. .N.Y..GN.- -......M.. 177
L60_(Portugal-1960) .TEI.NR..E L.YLDKK... .V...KSVDS .T.......S ....N..... .V....I..N .I-------- ---------- ---------- 151
OURT_88/3_(Portugal-1988) .TEI.NR..E L.YLDKK... .V...KSVDS .T.......S ....N..... .V....I..N .I.ND..GD. .N.Y..GN.- -......M.. 157
NHV_(Portugal-1968) .TEI.NR..E L.YLDKK... .V...KSVDS .T.......S ....N..... .V....I..N .I.ND..GD. .N.Y..GN.- -......M.. 157
Conservation
Ghana2022-35_EP402R NTISTSNETT LINCTYLTLS -SNYFYTFFK LYYIPLSIII GITISILLIS IITFLSLR-K RKKHVEEIES PPPESNEEEQ CQHDDTTSIH 263
ASFV-G_(Georgia-2007) .... -......... ....-. 263
BA71V_(Spain-1971) ....SL...E N....NPI.K YQ..LS.--- .F..IIF.VS .LI.G.F-.. ..SV..I.R. ....SESN..D IS........ 263
Benin_97/1_(Benin-1997) ....SL...E N....NPI.K YQ..LS.--- .F..IIF.VS .LI.G.F-.. ..SV..I.R. ....SESN..D IS........ 263
L60_(Portugal-1960) ---.SL...E N....NPI.K YQ..LS.--- .F..IIF.VS .LI.G.F-.. ..SV..I.R. ....SESN..D IS........ 234
OURT_88/3_(Portugal-1988) ....SL...E N....NPI.K YQ..LS.--- .F..IIF.VS .LI.G.F-.. ..SV..I.R. ....SESN..D IS........ 243
NHV_(Portugal-1968) ....SL...E N....NPI.K YQ..LS.--- .F..IIF.VS .LI.G.F-.. ..SV..I.R. ....SESN..D IS........ 243
Conservation
Ghana2022-35_EP402R EPSPREP--- ---------- LLPKPYSRYQ YNTPIYYMRP STQPLNPFPV PKPCPPPKPC PPLKPCPPPK PCP------- ---------- 323
ASFV-G_(Georgia-2007) ....--- ---------- ....L ....P....... ...------- ---------- 323
BA71V_(Spain-1971) ....--- ---------- ....L ....P....... ...PPKPCSP PKPCRPPKPC 340
Benin_97/1_(Benin-1997) ....--- ---------- ....L ....P....... ...PPKPCSP PKPCRPPKPC 340
L60_(Portugal-1960) ....--- ---------- ....L ....P....... ...PPKPCSP PKPCRPPKPC 311
OURT_88/3_(Portugal-1988) ....ENHYFL SLTVVISIIH .FTTCVPQHN HS.HFP.LNH ARH-..HVLH .SHARH.NLS ST*------- ---------- ---------- 305
NHV_(Portugal-1968) ....ENHYFL SLTVVISIIH .FTTCVPQHN HS.HFP.LNH ARH-..HVLH .SHARH.NLS ST*------- ---------- ---------- 305
Conservation
Ghana2022-35_EP402R ---------- ---------- -----SAESY SPPKPLPSIP LLPNIPPLST QNISLIHVDR II* 361
ASFV-G_(Georgia-2007) ---------- ---------- -----..... 361
BA71V_(Spain-1971) PPPKPCPPPK PCPPPKPCPP SKPCP.P... 403
Benin_97/1_(Benin-1997) PPPKPCPPPK PCPPPKPCPP SKPCP.P... 403
L60_(Portugal-1960) PPPKPCPPPK PCPPPKPCPP SKPCP.P... 374
OURT_88/3_(Portugal-1988) ---------- ---------- ---------- ---------- ---------- ---------- --- 305
NHV_(Portugal-1968) ---------- ---------- ---------- ---------- ---------- ---------- --- 305
Conservation

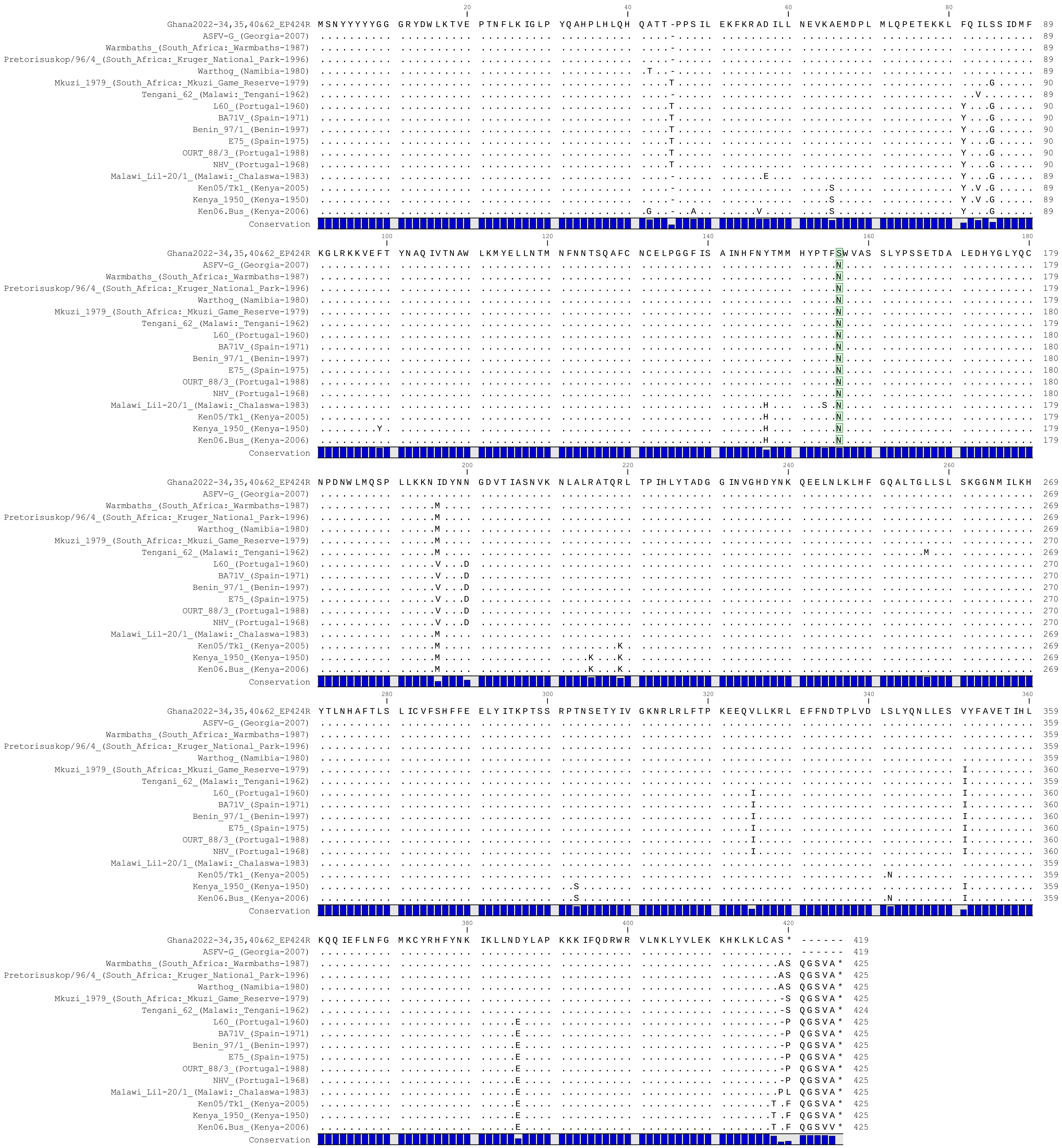

20 40 60 80
Ghana2022-34,35,40&62_EP424R MSNYYYYYGG GRYDWLKTVE PTNFLKIGLP YQAHPLHLQH QATT-PPSIL EKFKRADILL NEVKAEMDPL MLQPETEKKL FQILSSIDMF 89
ASFV-G_(Georgia-2007) ....-..... 89
Warmbaths_(South_Africa:_Warmbaths-1987) ....-..... 89
Pretorisuskop/96/4_(South_Africa:_Kruger_National_Park-1996) ....-..... 89
Warthog_(Namibia-1980) .... .T..-..... 89
Mkuzi_1979_(South_Africa:_Mkuzi_Game_Reserve-1979) ....T..... ....G..... 90
Tengani_62_(Malawi:_Tengani-1962) ....-..... ....V....... 89
L60_(Portugal-1960) ....T..... .... Y...G..... 90
BA71V_(Spain-1971) ....T..... .... Y...G..... 90
Benin_97/1_(Benin-1997) ....T..... .... Y...G..... 90
E75_(Spain-1975) ....T..... .... Y...G..... 90
OURT_88/3_(Portugal-1988) ....T..... .... Y...G..... 90
NHV_(Portugal-1968) ....T..... .... Y...G..... 90
Malawi_Lil-20/1_(Malawi:_Chalaswa-1983) ....-..... ......E... .... Y...G..... 89
Ken05/Tk1_(Kenya-2005) ....-..... ....S..... .... Y.V.G..... 89
Kenya_1950_(Kenya-1950) ....-..... ....S..... .... Y.V.G..... 89
Ken06.Bus_(Kenya-2006) .... .G..-..A.. ......V... ....S..... .... Y...G..... 89
Conservation
100 120 140 160 180
Ghana2022-34,35,40&62_EP424R KGLRKKVEFT YNAQIVTNAW LKMYELLNTM NFNNTSQAFC NCELPGGFIS AINHFNYTMM HYPTFSWVAS SLYPSSETDA LEDHYGLYQC 179
ASFV-G_(Georgia-2007) ....N.... 179
Warmbaths_(South_Africa:_Warmbaths-1987) ....N.... 179
Pretorisuskop/96/4_(South_Africa:_Kruger_National_Park-1996) ....N.... 179
Warthog_(Namibia-1980) ....N.... 179
Mkuzi_1979_(South_Africa:_Mkuzi_Game_Reserve-1979) ....N.... 180
Tengani_62_(Malawi:_Tengani-1962) ....N.... 179
L60_(Portugal-1960) ....N.... 180
BA71V_(Spain-1971) ....N.... 180
Benin_97/1_(Benin-1997) ....N.... 180
E75_(Spain-1975) ....N.... 180
OURT_88/3_(Portugal-1988) ....N.... 180
NHV_(Portugal-1968) ....N.... 180
Malawi_Lil-20/1_(Malawi:_Chalaswa-1983) ....H... ...S.N.... 179
Ken05/Tk1_(Kenya-2005) ....H... .....N.... 179
Kenya_1950_(Kenya-1950) ....Y. ....H... .....N.... 179
Ken06.Bus_(Kenya-2006) ....H... .....N.... 179
Conservation
200 220 240 260
Ghana2022-34,35,40&62_EP424R NPDNWLMQSP LLKKNIDYNN GDVTIASNVK NLALRATQRL TPIHLYTADG GINVGHDYNK QEELNLKLHF GQALTGLLSL SKGGNMILKH 269
ASFV-G_(Georgia-2007) 269
Warmbaths_(South_Africa:_Warmbaths-1987) ....M.... 269
Pretorisuskop/96/4_(South_Africa:_Kruger_National_Park-1996) ....M.... 269
Warthog_(Namibia-1980) ....M.... 269
Mkuzi_1979_(South_Africa:_Mkuzi_Game_Reserve-1979) ....M.... 270
Tengani_62_(Malawi:_Tengani-1962) ....M.... ....M... 269
L60_(Portugal-1960) ....V...D 270
BA71V_(Spain-1971) ....V...D 270
Benin_97/1_(Benin-1997) ....V...D 270
E75_(Spain-1975) ....V...D 270
OURT_88/3_(Portugal-1988) ....V...D 270
NHV_(Portugal-1968) ....V...D 270
Malawi_Lil-20/1_(Malawi:_Chalaswa-1983) ....M.... 269
Ken05/Tk1_(Kenya-2005) ....M.... ....K. 269
Kenya_1950_(Kenya-1950) ....M.... ....K...K. 269
Ken06.Bus_(Kenya-2006) ....M.... ....K...K. 269
Conservation
280 300 320 340 360
Ghana2022-34,35,40&62_EP424R YTLNHAFTLS LICVFSHFFE ELYITKPTSS RPTNSETYIV GKNRLRLFTP KEEQVLLKRL EFFNDTPLVD LSLYQNLLES VYFAVETIHL 359
ASFV-G_(Georgia-2007) 359
Warmbaths_(South_Africa:_Warmbaths-1987) 359
Pretorisuskop/96/4_(South_Africa:_Kruger_National_Park-1996) 359
Warthog_(Namibia-1980) 359
Mkuzi_1979_(South_Africa:_Mkuzi_Game_Reserve-1979) .... I......... 360
Tengani_62_(Malawi:_Tengani-1962) .... I......... 359
L60_(Portugal-1960) ....I..... .... I......... 360
BA71V_(Spain-1971) ....I..... .... I......... 360
Benin_97/1_(Benin-1997) ....I..... .... I......... 360
E75_(Spain-1975) ....I..... .... I......... 360
OURT_88/3_(Portugal-1988) ....I..... .... I......... 360
NHV_(Portugal-1968) ....I..... .... I......... 360
Malawi_Lil-20/1_(Malawi:_Chalaswa-1983) 359
Ken05/Tk1_(Kenya-2005) .... .N........ 359
Kenya_1950_(Kenya-1950) ....S....... .... I......... 359
Ken06.Bus_(Kenya-2006) ....S....... .... .N........ I......... 359
Conservation
380 400 420
Ghana2022-34,35,40&62_EP424R KQQIEFLNFG MKCYRHFYNK IKLLNDYLAP KKKIFQDRWR VLNKLYVLEK KHKLKLCAS* ------ 419
ASFV-G_(Georgia-2007) .... ------ 419
Warmbaths_(South_Africa:_Warmbaths-1987) ....AS QGSVA* 425
Pretorisuskop/96/4_(South_Africa:_Kruger_National_Park-1996) ....AS QGSVA* 425
Warthog_(Namibia-1980) ....AS QGSVA* 425
Mkuzi_1979_(South_Africa:_Mkuzi_Game_Reserve-1979) ....-S QGSVA* 425
Tengani_62_(Malawi:_Tengani-1962) ....-S QGSVA* 424
L60_(Portugal-1960) ....E.... ....-P QGSVA* 425
BA71V_(Spain-1971) ....E.... ....-P QGSVA* 425
Benin_97/1_(Benin-1997) ....E.... ....-P QGSVA* 425
E75_(Spain-1975) ....E.... ....-P QGSVA* 425
OURT_88/3_(Portugal-1988) ....E.... ....-P QGSVA* 425
NHV_(Portugal-1968) ....E.... ....-P QGSVA* 425
Malawi_Lil-20/1_(Malawi:_Chalaswa-1983) ....E.... ....PL QGSVA* 425
Ken05/Tk1_(Kenya-2005) ....E.... ....T.F QGSVA* 425
Kenya_1950_(Kenya-1950) ....E.... ....T.F QGSVA* 425
Ken06.Bus_(Kenya-2006) ....E.... ....T.F QGSVV* 425
Conservation

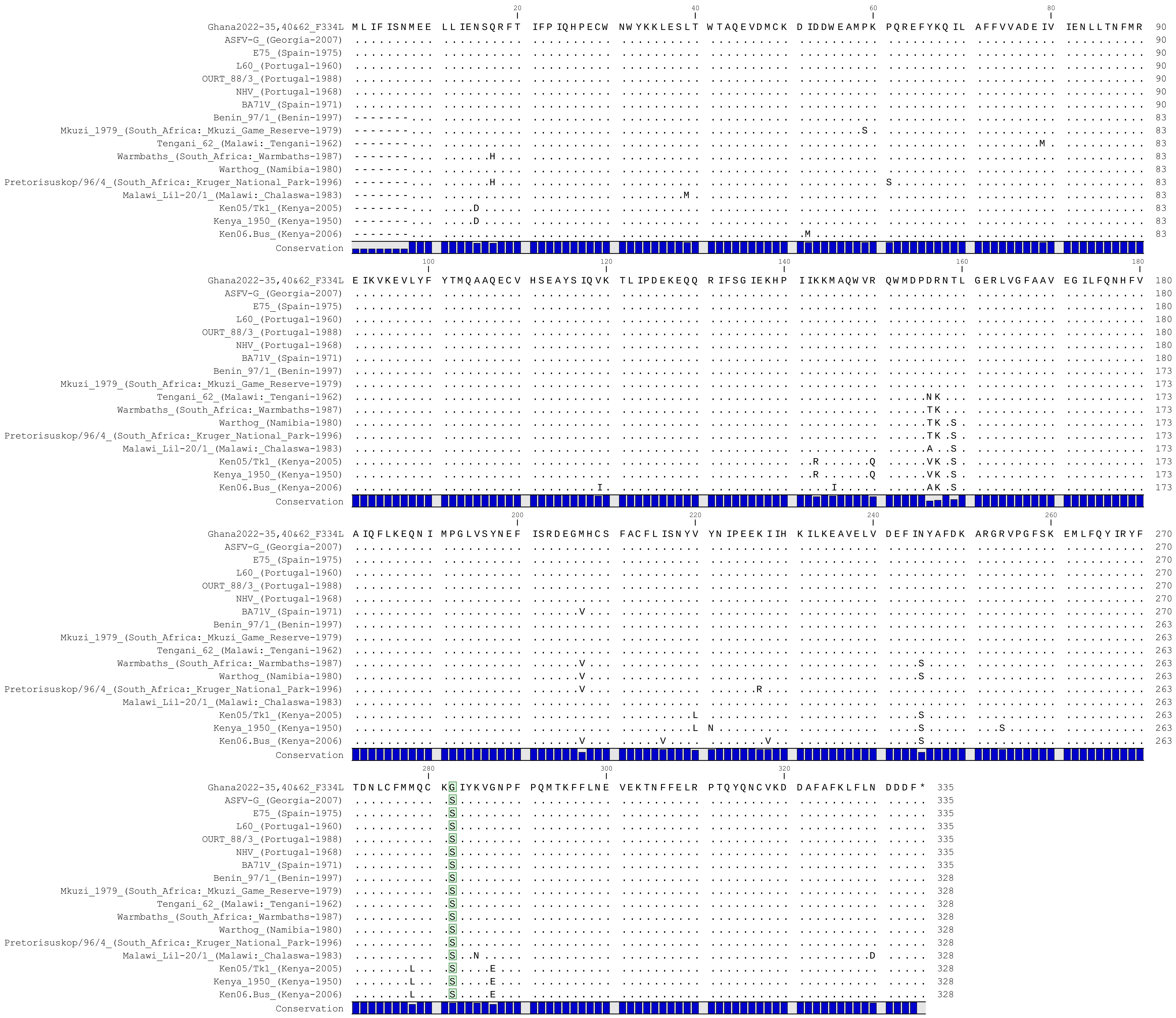
Ghana2022-35,40&62_F334L MLIFISNMEE LLIENSQRFT IFPIQHPECW NWYKKLESLT WTAQEVDMCK DIDDWEAMPK PQREFYKQIL AFFVVADEIV IENLLTNFMR 90
ASFV-G_(Georgia-2007)
E75_(Spain-1975)
L60_(Portugal-1960)
OURT_88/3_(Portugal-1988)
NHV_(Portugal-1968)
BA71V_(Spain-1971)
Benin_97/1_(Benin-1997)
Mkuzi_1979_(South_Africa:_Mkuzi_Game_Reserve-1979)
Tengani_62_(Malawi:_Tengani-1962)
Warmbaths_(South_Africa:_Warmbaths-1987)
Warthog_(Namibia-1980)
Pretorisuskop/96/4_(South_Africa:_Kruger_National_Park-1996)
Malawi_Lil-20/1_(Malawi:_Chalaswa-1983)
Ken05/Tk1_(Kenya-2005)
Kenya_1950_(Kenya-1950)
Ken06.Bus_(Kenya-2006)
Conservation
Ghana2022-35,40&62_F334L EIKVKEVLYF YTMQAAQECV HSEAYSIQVK TLIPDEKEQQ RIFSGIEKHP IIKKMAQWVR QWMDPDRNTL GERLVGFAAV EGILFQNHFV 180
Ghana2022-35,40&62_F334L AIQFLKEQNI MPGLVSYNEF ISRDEGMHCS FACFLISNYV YNIPEEKIIH KILKEAVELV DEFINYAFDK ARGRVPGFSK EMLFQYIRYF 270
Ghana2022-35,40&62_F334L TDNLCFMMQC KGIYKVGNPF PQMTKFFLNE VEKTNFFELR PTQYQNCVKD DAFAFKLFLN DDDF* 335

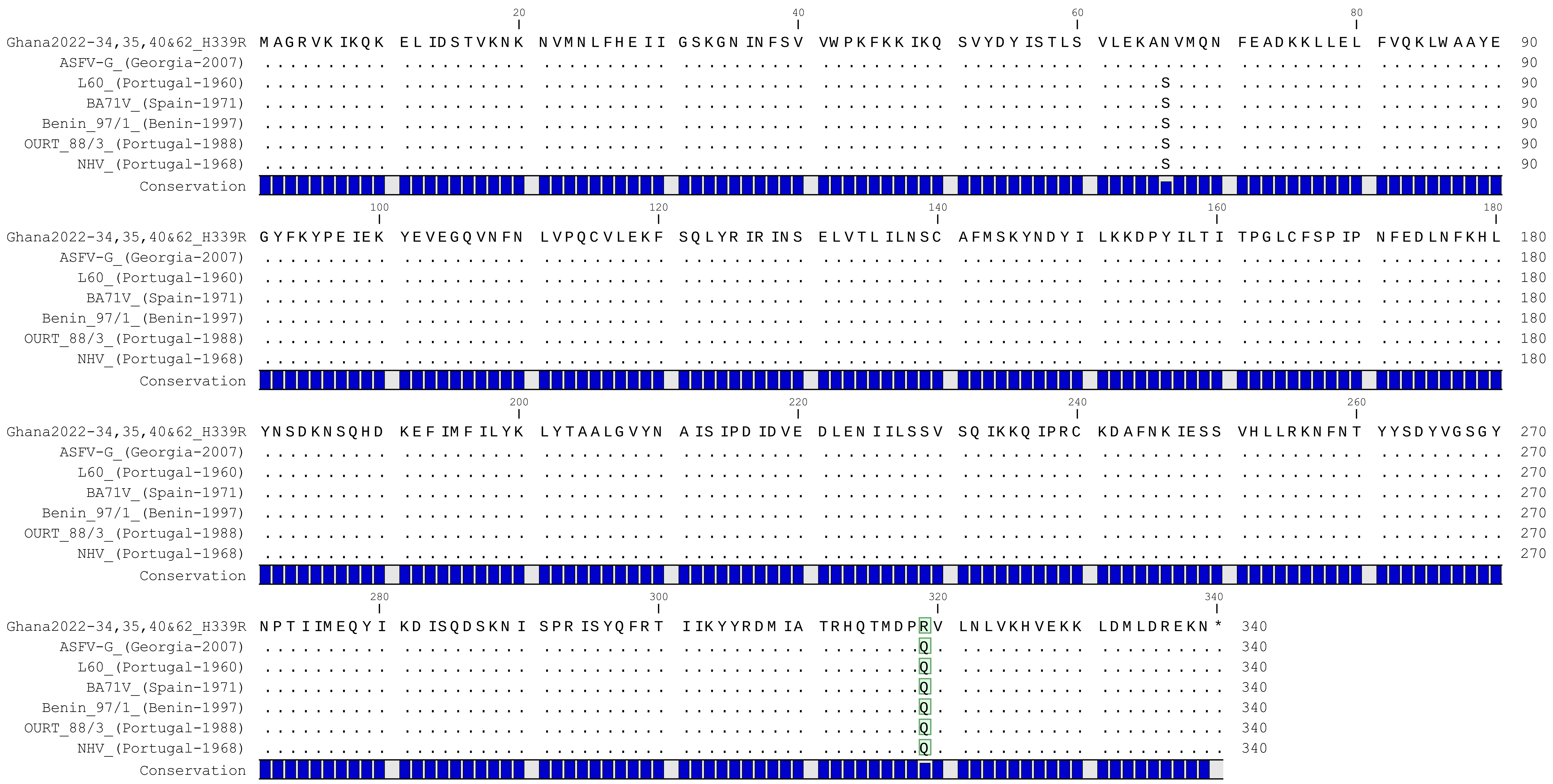

20 40 60 80
Ghana2022-34,35,40&62_H339R MAGRVKIKQK ELIDSTVKNK NVMNLFHEII GSKGNINFSV VWPKFKKIKQ SVYDYISTLS VLEKANVMQN FEADKKLLEL FVQKLWAAYE 90
ASFV-G_(Georgia-2007) 90
L60_(Portugal-1960) ....S.... 90
BA71V_(Spain-1971) ....S.... 90
Benin_97/1_(Benin-1997) ....S.... 90
OURT_88/3_(Portugal-1988) ....S.... 90
NHV_(Portugal-1968) ....S.... 90
Conservation
100 120 140 160 180
Ghana2022-34,35,40&62_H339R GYFKYPEIEK YEVEGQVNFN LVPQCVLEKF SQLYRIRINS ELVTLILNSC AFMSKYNDYI LKKDPYILTI TPGLCFSPIP NFEDLNFKHL 180
ASFV-G_(Georgia-2007) 180
L60_(Portugal-1960) 180
BA71V_(Spain-1971) 180
Benin_97/1_(Benin-1997) 180
OURT_88/3_(Portugal-1988) 180
NHV_(Portugal-1968) 180
Conservation
200 220 240 260
Ghana2022-34,35,40&62_H339R YNSDKNSQHD KEFIMFILYK LYTAALGVYN AISIPDIDVE DLENIILSSV SQIKKQIPRC KDAFNKIESS VHLLRKNFNT YYSDYVGSGY 270
ASFV-G_(Georgia-2007) 270
L60_(Portugal-1960) 270
BA71V_(Spain-1971) 270
Benin_97/1_(Benin-1997) 270
OURT_88/3_(Portugal-1988) 270
NHV_(Portugal-1968) 270
Conservation
280 300 320 340
Ghana2022-34,35,40&62_H339R NPTIIMEQYI KDISQDSKNI SPRISYQFRT IIKYYRDMIA TRHQTMDPRV LNLVKHVEKK LDMLDREKN* 340
ASFV-G_(Georgia-2007) ....Q. 340
L60_(Portugal-1960) ....Q. 340
BA71V_(Spain-1971) ....Q. 340
Benin_97/1_(Benin-1997) ....Q. 340
OURT_88/3_(Portugal-1988) ....Q. 340
NHV_(Portugal-1968) ....Q. 340
Conservation

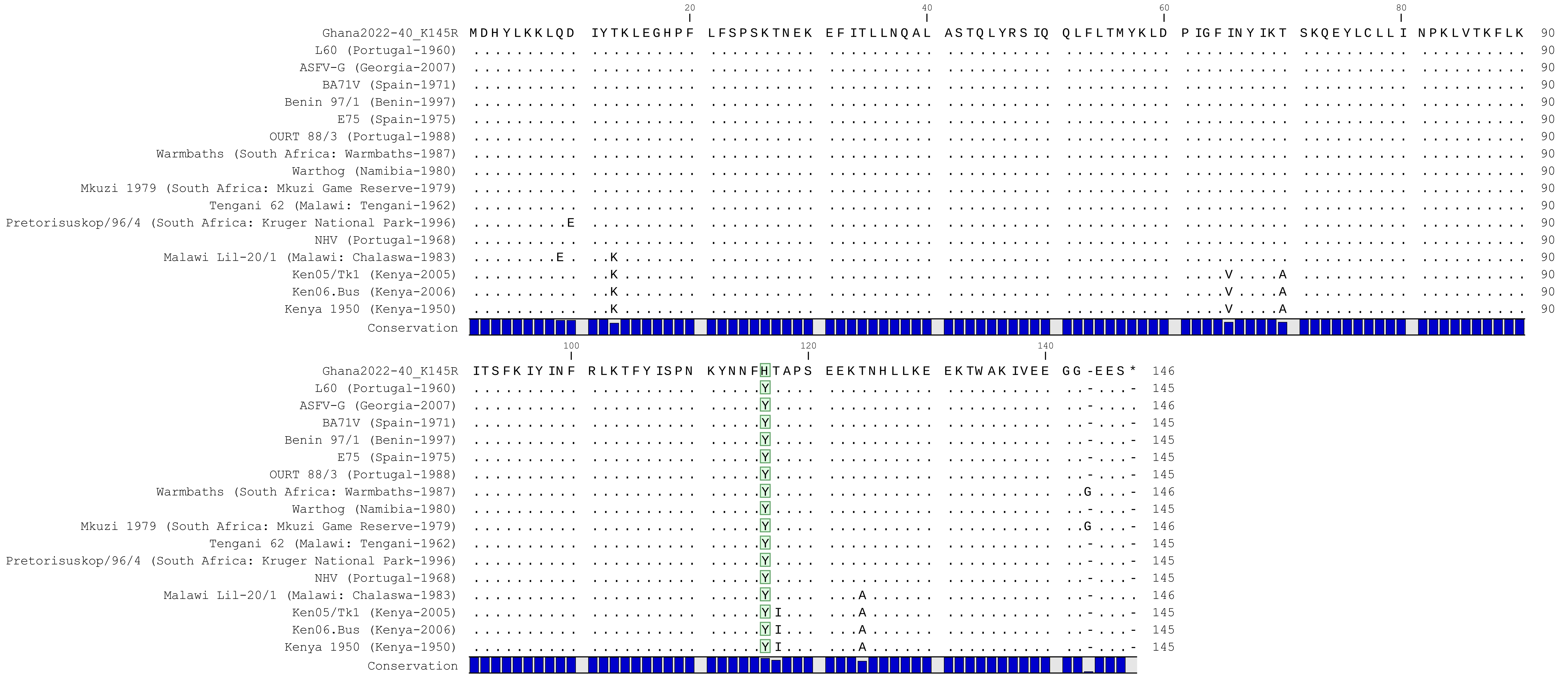

20
40
60
80
Ghana2022-40_K145R MDHYLKKLQD IYTKLEGHPF LFSPSKTNEK EFITLLNQAL ASTQLYRSIQ QLFLTMYKLD PIGFINYIKT SKQEYLCLLI NPKLVTKFLK 90
L60 (Portugal-1960)
ASFV-G (Georgia-2007)
BA71V (Spain-1971)
Benin 97/1 (Benin-1997)
E75 (Spain-1975)
OURT 88/3 (Portugal-1988)
Warmbaths (South Africa: Warmbaths-1987)
Warthog (Namibia-1980)
Mkuzi 1979 (South Africa: Mkuzi Game Reserve-1979)
Tengani 62 (Malawi: Tengani-1962)
Pretorisuskop/96/4 (South Africa: Kruger National Park-1996)
NHV (Portugal-1968)
Malawi Lil-20/1 (Malawi: Chalaswa-1983)
Ken05/Tk1 (Kenya-2005)
Ken06.Bus (Kenya-2006)
Kenya 1950 (Kenya-1950)
Conservation
100
120
140
Ghana2022-40_K145R ITSFKIYINF RLKTFYISPN KYNNFHTAPS EEKTNHLLKE EKTWAKIVEE GG-EES* 146
L60 (Portugal-1960) 145
ASFV-G (Georgia-2007) 146
BA71V (Spain-1971) 145
Benin 97/1 (Benin-1997) 145
E75 (Spain-1975) 145
OURT 88/3 (Portugal-1988) 145
Warmbaths (South Africa: Warmbaths-1987) 146
Warthog (Namibia-1980) 145
Mkuzi 1979 (South Africa: Mkuzi Game Reserve-1979) 146
Tengani 62 (Malawi: Tengani-1962) 145
Pretorisuskop/96/4 (South Africa: Kruger National Park-1996) 145
NHV (Portugal-1968) 145
Malawi Lil-20/1 (Malawi: Chalaswa-1983) 146
Ken05/Tk1 (Kenya-2005) 145
Ken06.Bus (Kenya-2006) 145
Kenya 1950 (Kenya-1950) 145
Conservation

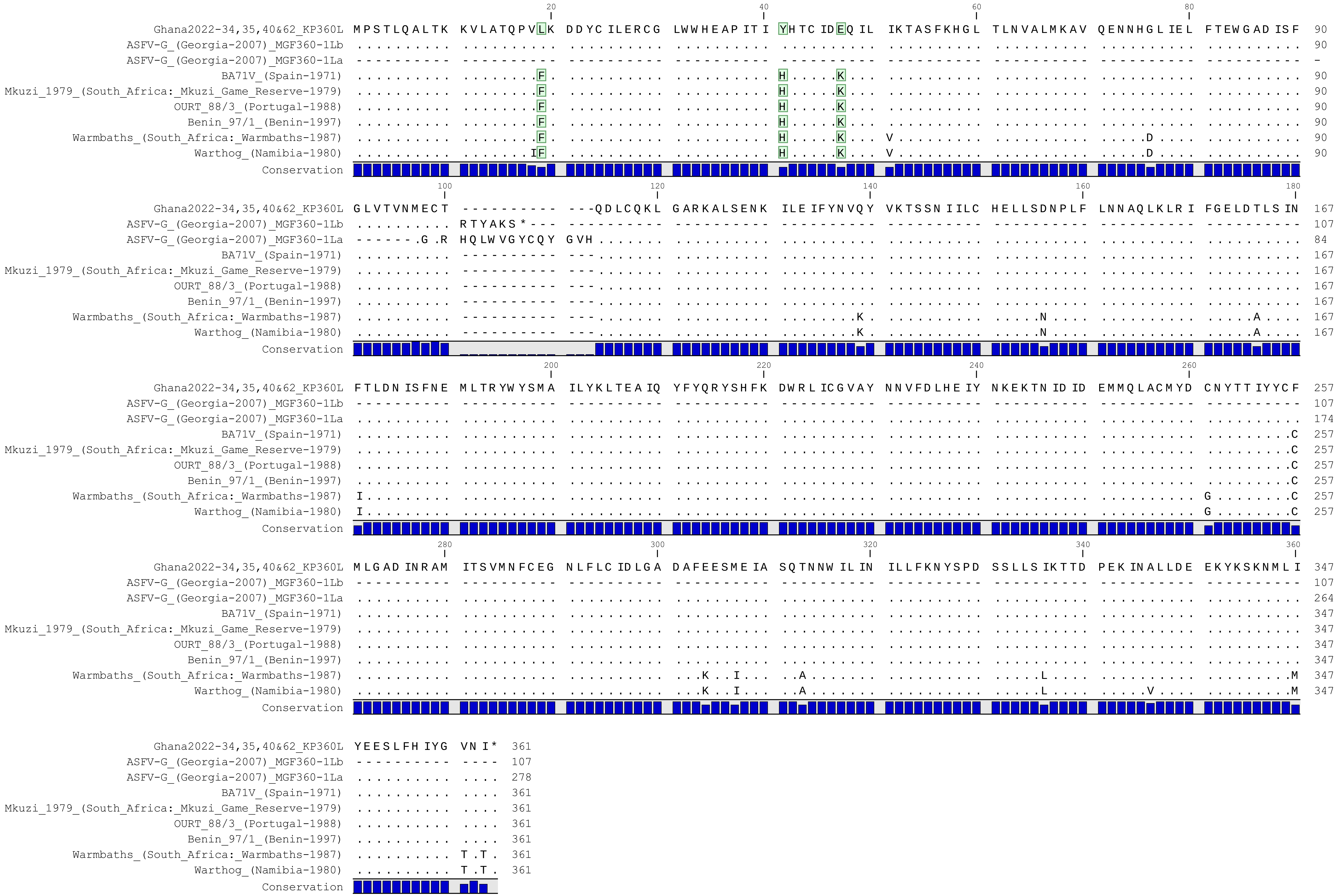

20 40 60 80
Ghana2022-34,35,40&62_KP360L MPSTLQALTK KVLATQPVLK DDYCILERCG LWWHEAPITI YHTCIDEQIL IKTASFKHGL TLNVALMKAV QENNHGLIEL FTEWGADISF 90
ASFV-G_(Georgia-2007)_MGF360-1Lb 90
ASFV-G_(Georgia-2007)_MGF360-1La ---------- ---------- ---------- ---------- ---------- ---------- ---------- ---------- ---------- -
BA71V_(Spain-1971) ....F. .... H.....K... 90
Mkuzi_1979_(South_Africa:_Mkuzi_Game_Reserve-1979) ....F. .... H.....K... 90
OURT_88/3_(Portugal-1988) ....F. .... H.....K... 90
Benin_97/1_(Benin-1997) ....F. .... H.....K... 90
Warmbaths_(South_Africa:_Warmbaths-1987) ....F. .... H.....K... V......... ....D.... 90
Warthog_(Namibia-1980) ....IF. .... H.....K... V......... ....D.... 90
Conservation
100 120 140 160 180
Ghana2022-34,35,40&62_KP360L GLVTVNMECT ---------- ---QDLCQKL GARKALSENK ILEIFYNVQY VKTSSNIILC HELLSDNPLF LNNAQLKLRI FGELDTLSIN 167
ASFV-G_(Georgia-2007)_MGF360-1Lb .... RTYAKS*--- ---------- ---------- ---------- ---------- ---------- ---------- ---------- 107
ASFV-G_(Georgia-2007)_MGF360-1La ------.G.R HQLWVGYCQY GVH....... 84
BA71V_(Spain-1971) .... ---------- ---....... 167
Mkuzi_1979_(South_Africa:_Mkuzi_Game_Reserve-1979) .... ---------- ---....... 167
OURT_88/3_(Portugal-1988) .... ---------- ---....... 167
Benin_97/1_(Benin-1997) .... ---------- ---....... 167
Warmbaths_(South_Africa:_Warmbaths-1987) .... ---------- ---....... ....K. ....N.... ....A.... 167
Warthog_(Namibia-1980) .... ---------- ---....... ....K. ....N.... ....A.... 167
Conservation
200 220 240 260
Ghana2022-34,35,40&62_KP360L FTLDNISFNE MLTRYWYSMA ILYKLTEAIQ YFYQRYSHFK DWRLICGVAY NNVFDLHEIY NKEKTNIDID EMMQLACMYD CNYTTIYYCF 257
ASFV-G_(Georgia-2007)_MGF360-1Lb ---------- ---------- ---------- ---------- ---------- ---------- ---------- ---------- ---------- 107
ASFV-G_(Georgia-2007)_MGF360-1La 174
BA71V_(Spain-1971) ....C 257
Mkuzi_1979_(South_Africa:_Mkuzi_Game_Reserve-1979) ....C 257
OURT_88/3_(Portugal-1988) ....C 257
Benin_97/1_(Benin-1997) ....C 257
Warmbaths_(South_Africa:_Warmbaths-1987) I......... .... G........C 257
Warthog_(Namibia-1980) I......... .... G........C 257
Conservation
280 300 320 340 360
Ghana2022-34,35,40&62_KP360L MLGADINRAM ITSVMNFCEG NLFLCIDLGA DAFEESMEIA SQTNNWILIN ILLFKNYSPD SSLLSIKTTD PEKINALLDE EKYKSKNMLI 347
ASFV-G_(Georgia-2007)_MGF360-1Lb ---------- ---------- ---------- ---------- ---------- ---------- ---------- ---------- ---------- 107
ASFV-G_(Georgia-2007)_MGF360-1La 264
BA71V_(Spain-1971) 347
Mkuzi_1979_(South_Africa:_Mkuzi_Game_Reserve-1979) 347
OURT_88/3_(Portugal-1988) 347
Benin_97/1_(Benin-1997) 347
Warmbaths_(South_Africa:_Warmbaths-1987) ....K..I... ..A....... ....L.... ....M 347
Warthog_(Namibia-1980) ....K..I... ..A....... ....L.... .....V.... ....M 347
Conservation
Ghana2022-34,35,40&62_KP360L YEESLFHIYG VNI* 361
ASFV-G_(Georgia-2007)_MGF360-1Lb ---------- ---- 107
ASFV-G_(Georgia-2007)_MGF360-1La 278
BA71V_(Spain-1971) 361
Mkuzi_1979_(South_Africa:_Mkuzi_Game_Reserve-1979) 361
OURT_88/3_(Portugal-1988) 361
Benin_97/1_(Benin-1997) 361
Warmbaths_(South_Africa:_Warmbaths-1987) .... T.T. 361
Warthog_(Namibia-1980) .... T.T. 361
Conservation

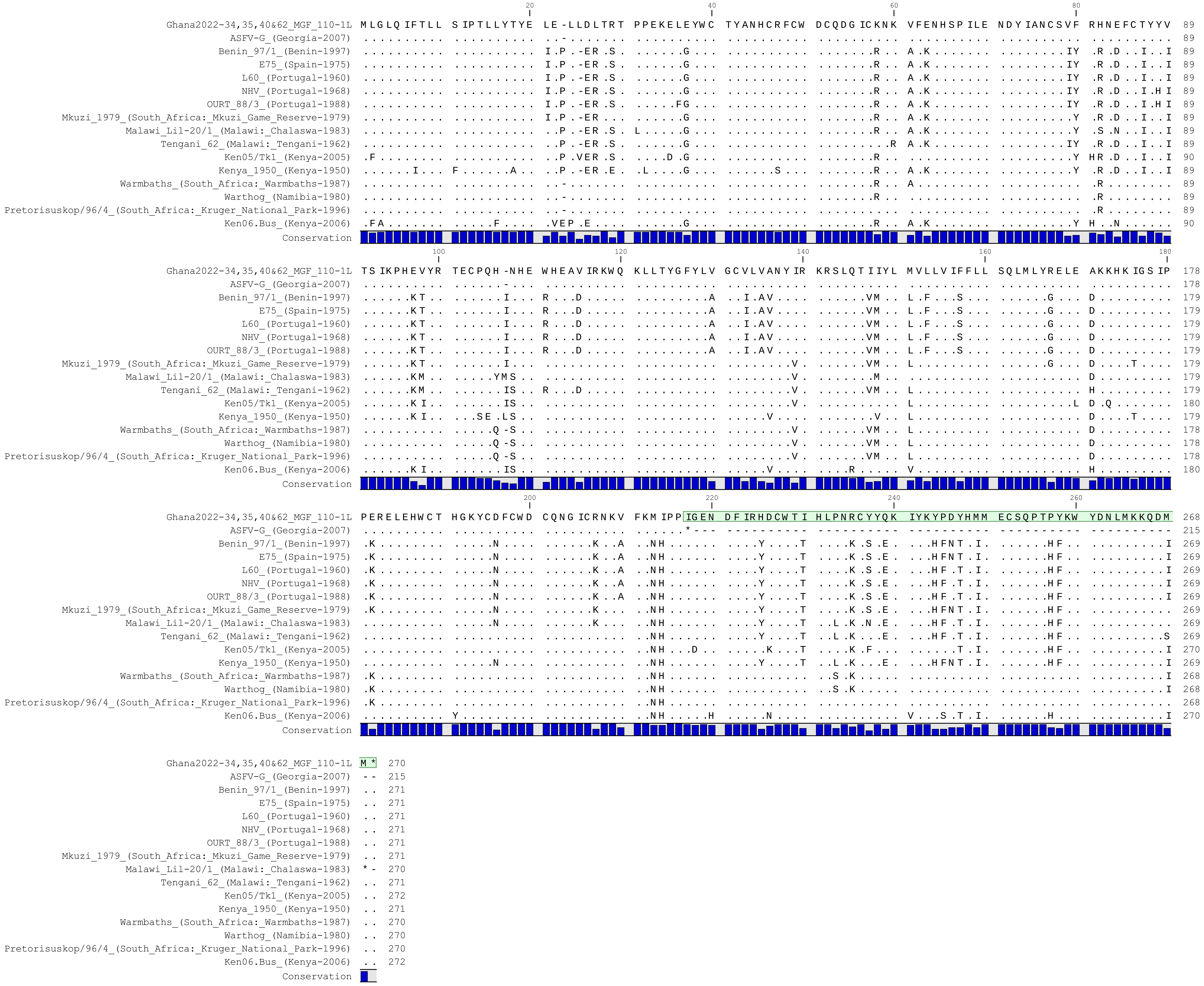
Ghana2022-34,35,40&62_MGF_110-1L
ASFV-G_(Georgia-2007)
Benin_97/1_(Benin-1997)
E75_(Spain-1975)
L60_(Portugal-1960)
NHV_(Portugal-1968)
OURT_88/3_(Portugal-1988)
Mkuzi_1979_(South_Africa:_Mkuzi_Game_Reserve-1979)
Malawi_Lil-20/1_(Malawi:_Chalaswa-1983)
Tengani_62_(Malawi:_Tengani-1962)
Ken05/Tk1_(Kenya-2005)
Kenya_1950_(Kenya-1950)
Warmbaths_(South_Africa:_Warmbaths-1987)
Warthog_(Namibia-1980)
Pretorisuskop/96/4_(South_Africa:_Kruger_National_Park-1996)
Ken06.Bus_(Kenya-2006)
Conservation
MLGLQIFTLL SIPTLLYTYE LE-LLDLTRT PPEKELEYWC TYANHCRFCW DCQDGICKNK VFENHSPILE NDYIANCSVF RHNEFCTYYV 89
TSIKPHEVYR TECPQH-NHE WHEAVIRKWQ KLLTYGFYLV GCVLVANYIR KRSLQTIIYL MVLLVIFFLL SQLMLYRELE AKKHKIGSIP 178
PERELEHWCT HGKYCDFCWD CQNGICRNKV FKMIPPIGEN DFIRHDCWTI HLPNRCYYQK IYKYPDYHMM ECSQPTPYKW YDNLMKKQDM 268
M* 270

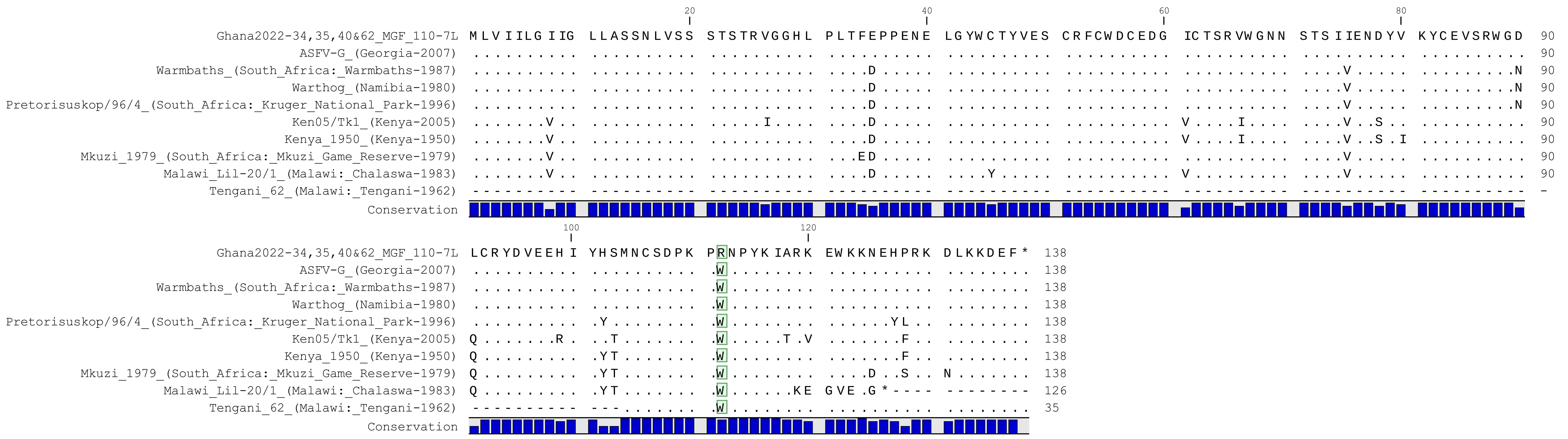
20
40
60
80
Ghana2022-34,35,40&62_MGF_110-7L MLVIILGIIG LLASSNLVSS STSTRVGGHL PLTFEPPENE LGYWCTYVES CRFCWDCEDG ICTSRVWGNN STSIIENDYV KYCEVSRWGD 90
ASFV-G_(Georgia-2007) 90
Warmbaths_(South_Africa:_Warmbaths-1987) 90
Warthog_(Namibia-1980) 90
Pretorisuskop/96/4_(South_Africa:_Kruger_National_Park-1996) 90
Ken05/Tk1_(Kenya-2005) 90
Kenya_1950_(Kenya-1950) 90
Mkuzi_1979_(South_Africa:_Mkuzi_Game_Reserve-1979) 90
Malawi_Lil-20/1_(Malawi:_Chalaswa-1983) 90
Tengani_62_(Malawi:_Tengani-1962) -
Conservation
100
120
Ghana2022-34,35,40&62_MGF_110-7L LCRYDVEEHI YHSMNCSDPK PRNPYKIARK EWKKNEHPRK DLKKDEF* 138
ASFV-G_(Georgia-2007) 138
Warmbaths_(South_Africa:_Warmbaths-1987) 138
Warthog_(Namibia-1980) 138
Pretorisuskop/96/4_(South_Africa:_Kruger_National_Park-1996) 138
Ken05/Tk1_(Kenya-2005) 138
Kenya_1950_(Kenya-1950) 138
Mkuzi_1979_(South_Africa:_Mkuzi_Game_Reserve-1979) 138
Malawi_Lil-20/1_(Malawi:_Chalaswa-1983) 126
Tengani_62_(Malawi:_Tengani-1962) 35
Conservation

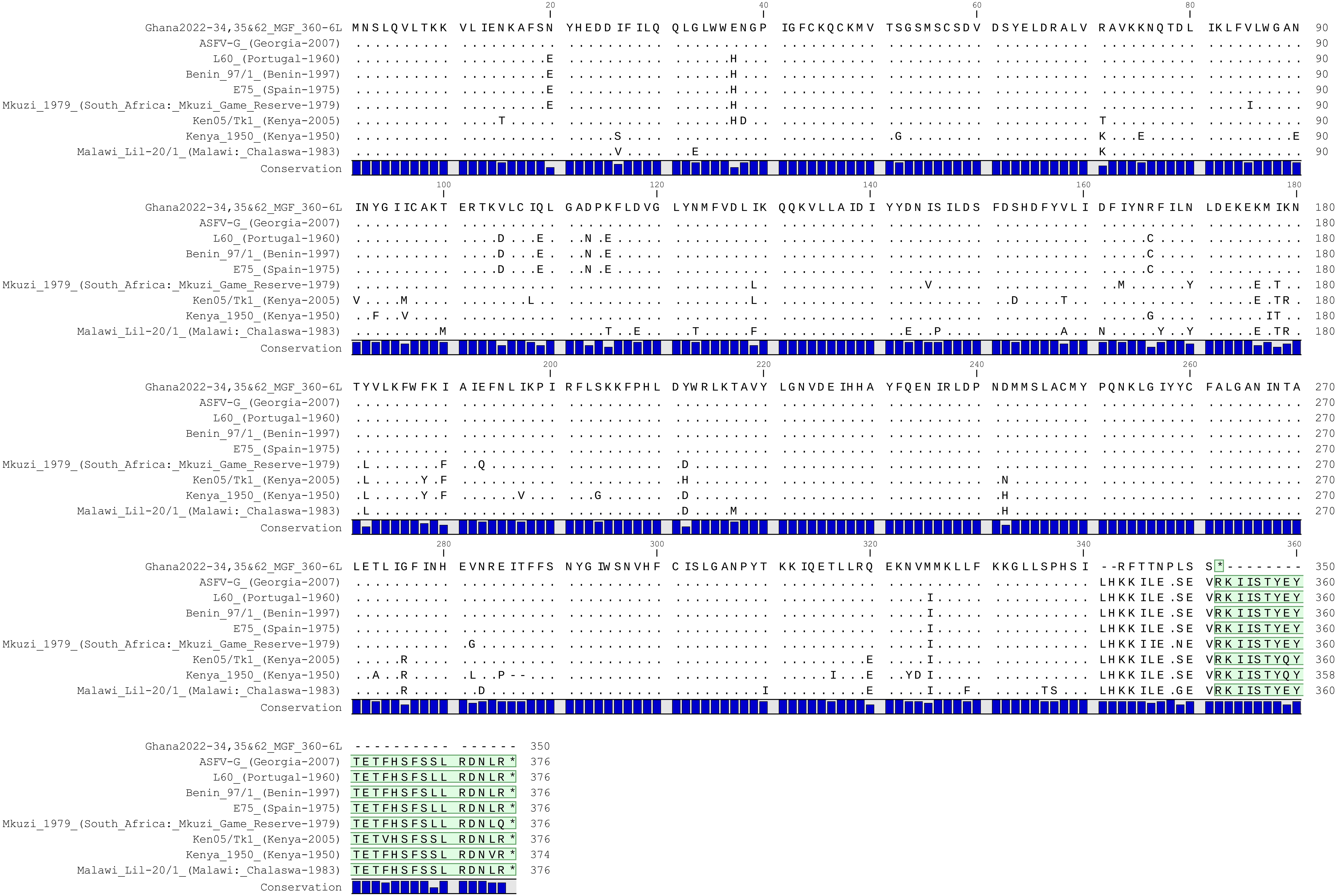

Ghana2022-34,35&62_MGF_360-6L MNSLQVLTKK VLIENKAFSN YHEDDIFILQ QLGLWWENGP IGFCKQCKMV TSGSMSCSDV DSYELDRALV RAVKKNQTDL IKLFVLWGAN 90
ASFV-G_(Georgia-2007) 90
L60_(Portugal-1960) ....E ....H... 90
Benin_97/1_(Benin-1997) ....E ....H... 90
E75_(Spain-1975) ....E ....H... 90
Mkuzi_1979_(South_Africa:_Mkuzi_Game_Reserve-1979) ....E ....H... ....I..... 90
Ken05/Tk1_(Kenya-2005) ....T..... ....HD.. .... T......... 90
Kenya_1950_(Kenya-1950) ....S.... .... .G........ .... K...E..... ....E 90
Malawi_Lil-20/1_(Malawi:_Chalaswa-1983) ....V.... ..E....... .... K......... 90
Conservation
Ghana2022-34,35&62_MGF_360-6L INYGIICAKT ERTKVLCIQL GADPKFLDVG LYNMFVDLIK QQKVLLAIDI YYDNISILDS FDSHDFYVLI DFIYNRFILN LDEKEKMIKN 180
ASFV-G_(Georgia-2007) 180
L60_(Portugal-1960) ....D...E. ..N.E..... ....C.... 180
Benin_97/1_(Benin-1997) ....D...E. ..N.E..... ....C.... 180
E75_(Spain-1975) ....D...E. ..N.E..... ....C.... 180
Mkuzi_1979_(South_Africa:_Mkuzi_Game_Reserve-1979) ....L. ....V..... ....M......Y .....E.T.. 180
Ken05/Tk1_(Kenya-2005) V....M.... ....L.. ....L. ....D....T.. ....E.TR. 180
Kenya_1950_(Kenya-1950) ..F..V.... ....G.... ......IT.. 180
Malawi_Lil-20/1_(Malawi:_Chalaswa-1983) ....M ....T..E.. ..T.....F. ....E..P.... ....A.. N.....Y..Y .....E.TR. 180
Conservation
Ghana2022-34,35&62_MGF_360-6L TYVLKFWFKI AIEFNLIKPI RFLSKKFPHL DYWRLKTAVY LGNVDEIHHA YFQENIRLDP NDMMSLACMY PQNKLGIYYC FALGANINTA 270
ASFV-G_(Georgia-2007) 270
L60_(Portugal-1960) 270
Benin_97/1_(Benin-1997) 270
E75_(Spain-1975) 270
Mkuzi_1979_(South_Africa:_Mkuzi_Game_Reserve-1979) .L.......F ..Q....... .... .D........ 270
Ken05/Tk1_(Kenya-2005) .L.....Y.F .... .H........ .... .N........ 270
Kenya_1950_(Kenya-1950) .L.....Y.F ......V... ...G...... .D........ .... .H........ 270
Malawi_Lil-20/1_(Malawi:_Chalaswa-1983) .L........ .... .D....M... .... .H........ 270
Conservation
Ghana2022-34,35&62_MGF_360-6L LETLIGFINH EVNREITFFS NYGIWSNVHF CISLGANPYT KKIQETLLRQ EKNVMMKLLF KKGLLSPHSI --RFTTNPLS S*-------- 350
ASFV-G_(Georgia-2007) .... LHKKILE.SE VRKIISTYEY 360
L60_(Portugal-1960) ....I..... .... LHKKILE.SE VRKIISTYEY 360
Benin_97/1_(Benin-1997) ....I..... .... LHKKILE.SE VRKIISTYEY 360
E75_(Spain-1975) ....I..... .... LHKKILE.SE VRKIISTYEY 360
Mkuzi_1979_(South_Africa:_Mkuzi_Game_Reserve-1979) .... .G........ ....I..... .... LHKKIIE.NE VRKIISTYEY 360
Ken05/Tk1_(Kenya-2005) .....R.... ....E ....I..... .... LHKKILE.SE VRKIISTYQY 360
Kenya_1950_(Kenya-1950) ..A..R.... .L..P--... ....I...E ..YDI..... .... LHKKILE.SE VRKIISTYQY 358
Malawi_Lil-20/1_(Malawi:_Chalaswa-1983) .....R.... ..D....... ....I ....E ....I...F. .....TS... LHKKILE.GE VRKIISTYEY 360
Conservation
Ghana2022-34,35&62_MGF_360-6L ---------- ------ 350
ASFV-G_(Georgia-2007) TETFHSFSSL RDNLR* 376
L60_(Portugal-1960) TETFHSFSLL RDNLR* 376
Benin_97/1_(Benin-1997) TETFHSFSLL RDNLR* 376
E75_(Spain-1975) TETFHSFSLL RDNLR* 376
Mkuzi_1979_(South_Africa:_Mkuzi_Game_Reserve-1979) TETFHSFSLL RDNLQ* 376
Ken05/Tk1_(Kenya-2005) TETVHSFSSL RDNLR* 376
Kenya_1950_(Kenya-1950) TETFHSFSSL RDNVR* 374
Malawi_Lil-20/1_(Malawi:_Chalaswa-1983) TETFHSFSSL RDNLR* 376
Conservation

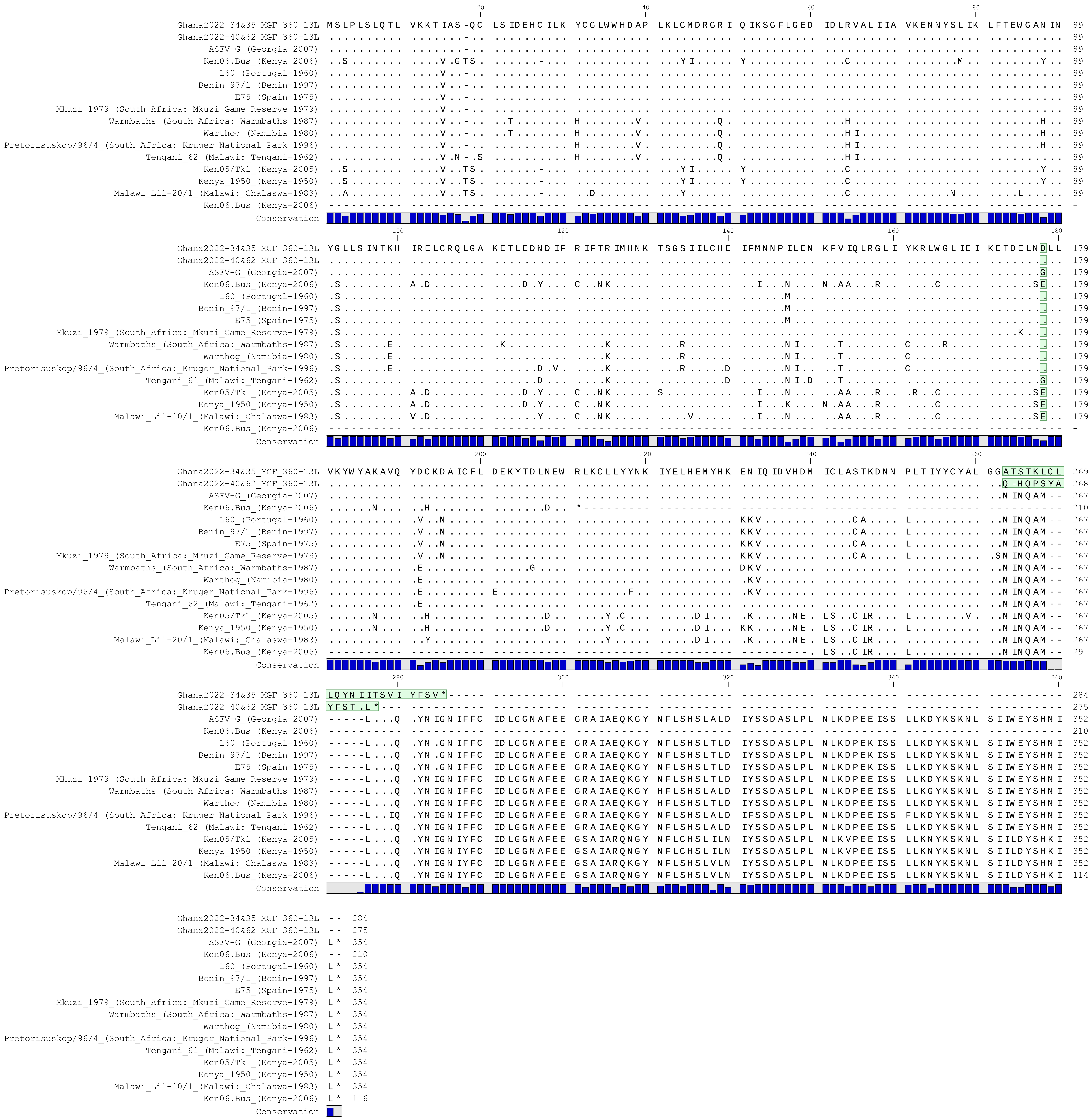
Ghana2022-34&35_MGF_360-13L
Ghana2022-40&62_MGF_360-13L
ASFV-G_(Georgia-2007)
Ken06.Bus_(Kenya-2006)
L60_(Portugal-1960)
Benin_97/1_(Benin-1997)
E75_(Spain-1975)
Mkuzi_1979_(South_Africa:_Mkuzi_Game_Reserve-1979)
Warmbaths_(South_Africa:_Warmbaths-1987)
Warthog_(Namibia-1980)
Pretorisuskop/96/4_(South_Africa:_Kruger_National_Park-1996)
Tengani_62_(Malawi:_Tengani-1962)
Ken05/Tk1_(Kenya-2005)
Kenya_1950_(Kenya-1950)
Malawi_Lil-20/1_(Malawi:_Chalaswa-1983)
Ken06.Bus_(Kenya-2006)
Conservation

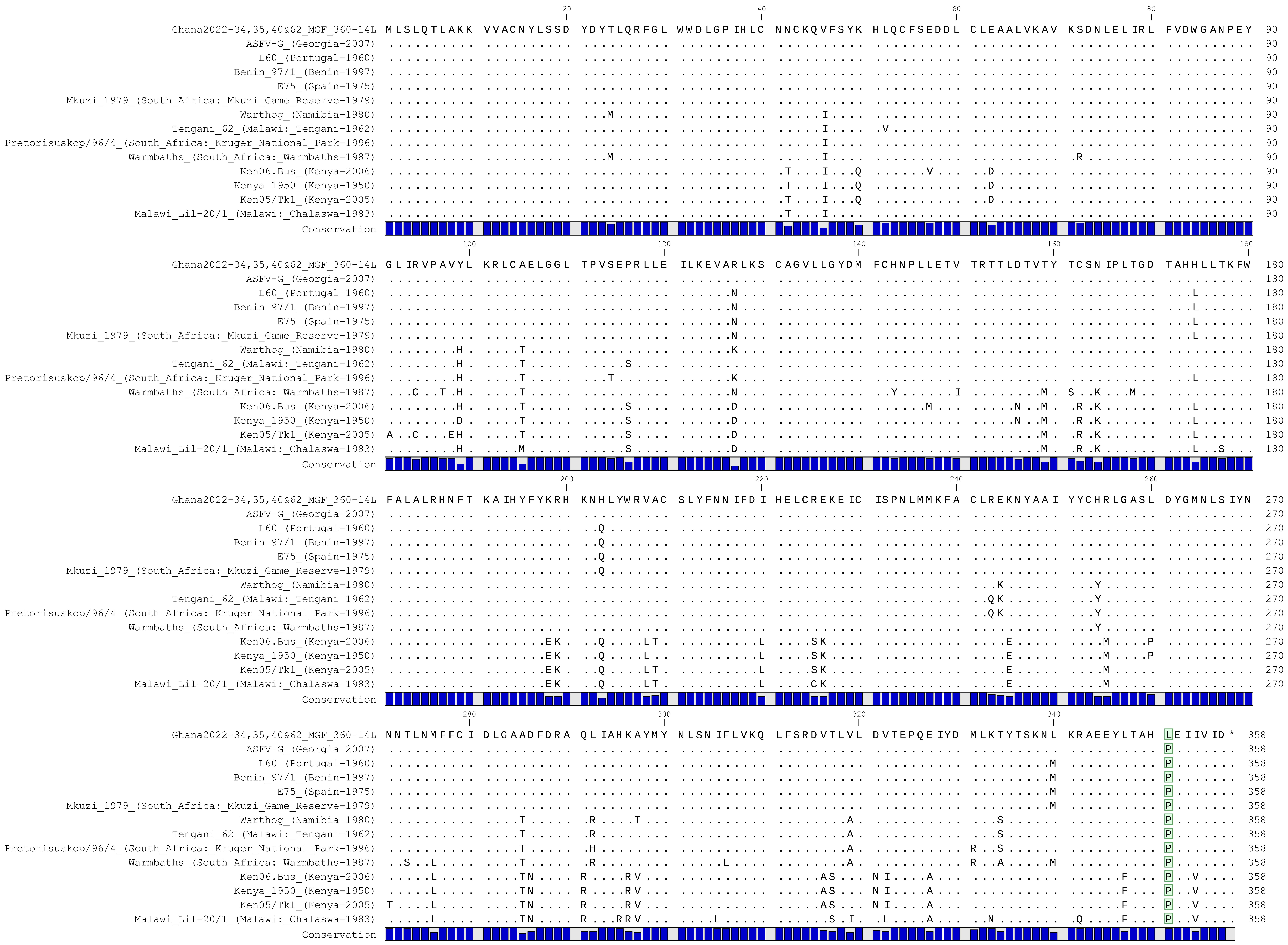
Ghana2022-34,35,40&62_MGF_360-14L
ASFV-G_(Georgia-2007)
L60_(Portugal-1960)
Benin_97/1_(Benin-1997)
E75_(Spain-1975)
Mkuzi_1979_(South_Africa:_Mkuzi_Game_Reserve-1979)
Warthog_(Namibia-1980)
Tengani_62_(Malawi:_Tengani-1962)
Pretorisuskop/96/4_(South_Africa:_Kruger_National_Park-1996)
Warmbaths_(South_Africa:_Warmbaths-1987)
Ken06.Bus_(Kenya-2006)
Kenya_1950_(Kenya-1950)
Ken05/Tk1_(Kenya-2005)
Malawi_Lil-20/1_(Malawi:_Chalaswa-1983)
Conservation
MLSLQTLAKK VVACNYLSSD YDYTLQRFGL WWDLGPIHLC NNCKQVFSYK HLQCFSEDDL CLEAALVKAV KSDNLELIRL FVDWGANPEY 90
GLIRVPAVYL KRLCAELGGL TPVSEPRLLE ILKEVARLKS CAGVLLGYDM FCHNPLLETV TRTTLDTVTY TCSNIPLTGD TAHHLLTKFW 180
FALALRHNFT KAIHYFYKRH KNHLYWRVAC SLYFNNIFDI HELCREKEIC ISPNLMMKFA CLREKNYAAI YYCHRLGASL DYGMNLSIYN 270
NNTLNMFFCI DLGAADFDRA QLIAHKAYMY NLSNIFLVKQ LFSRDVTLVL DVTEPQEIYD MLKTYTSKNL KRAEEYLTAH LEIIVID* 358

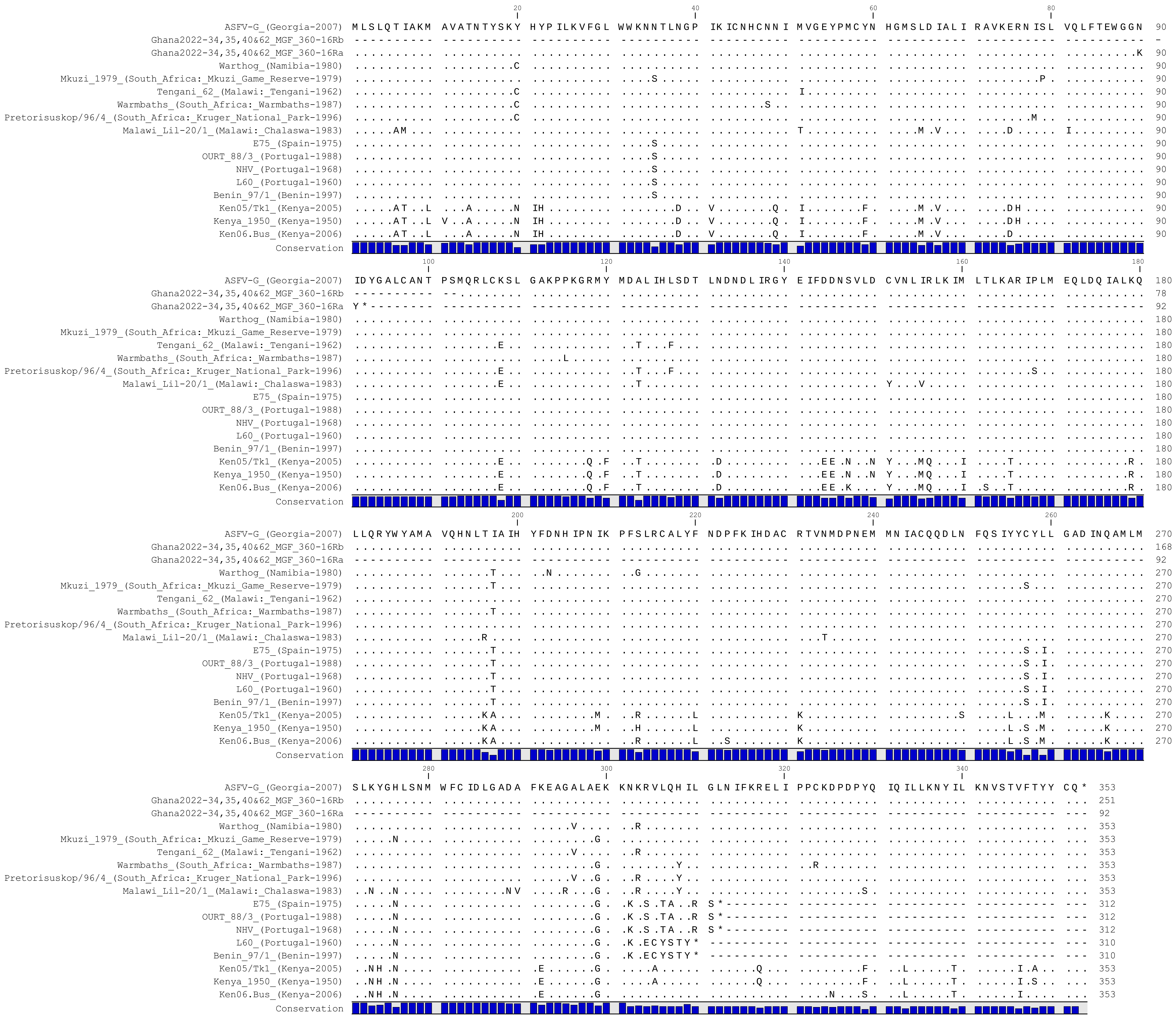
ASFV-G_(Georgia-2007)
Ghana2022-34,35,40&62_MGF_360-16Rb
Ghana2022-34,35,40&62_MGF_360-16Ra
Warthog_(Namibia-1980)
Mkuzi_1979_(South_Africa:_Mkuzi_Game_Reserve-1979)
Tengani_62_(Malawi:_Tengani-1962)
Warmbaths_(South_Africa:_Warmbaths-1987)
Pretorisuskop/96/4_(South_Africa:_Kruger_National_Park-1996)
Malawi_Lil-20/1_(Malawi:_Chalaswa-1983)
E75_(Spain-1975)
OURT_88/3_(Portugal-1988)
NHV_(Portugal-1968)
L60_(Portugal-1960)
Benin_97/1_(Benin-1997)
Ken05/Tk1_(Kenya-2005)
Kenya_1950_(Kenya-1950)
Ken06.Bus_(Kenya-2006)
Conservation

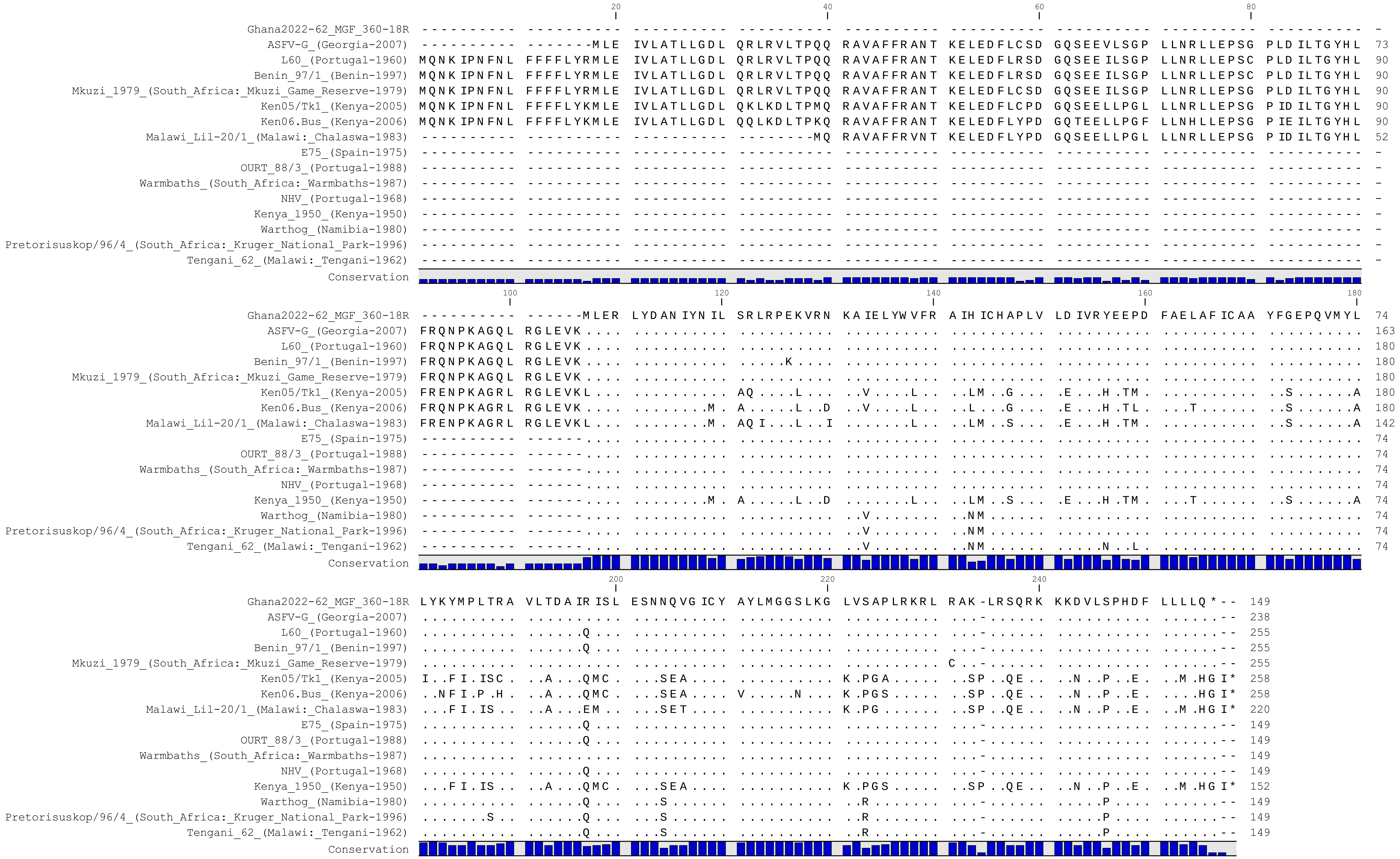

Ghana2022-62_MGF_360-18R
ASFV-G_(Georgia-2007)
L60_(Portugal-1960)
Benin_97/1_(Benin-1997)
Mkuzi_1979_(South_Africa:_Mkuzi_Game_Reserve-1979)
Ken05/Tk1_(Kenya-2005)
Ken06.Bus_(Kenya-2006)
Malawi_Lil-20/1_(Malawi:_Chalaswa-1983)
E75_(Spain-1975)
OURT_88/3_(Portugal-1988)
Warmbaths_(South_Africa:_Warmbaths-1987)
NHV_(Portugal-1968)
Kenya_1950_(Kenya-1950)
Warthog_(Namibia-1980)
Pretorisuskop/96/4_(South_Africa:_Kruger_National_Park-1996)
Tengani_62_(Malawi:_Tengani-1962)
Conservation
20 40 60 80
ASFV-G_(Georgia-2007) MLE IVLATLLGDL QRLRVLTPQQ RAVAFFRANT KELEDFLCSD GQSEEVLSGP LLNRLLEPSG PLDILTGYHL 73
L60_(Portugal-1960) MQNKIPNFNL FFFFLYRMLE IVLATLLGDL QRLRVLTPQQ RAVAFFRANT KELEDFLRSD GQSEEILSGP LLNRLLEPSC PLDILTGYHL 90
Benin_97/1_(Benin-1997) MQNKIPNFNL FFFFLYRMLE IVLATLLGDL QRLRVLTPQQ RAVAFFRANT KELEDFLRSD GQSEEILSGP LLNRLLEPSC PLDILTGYHL 90
Mkuzi_1979_(South_Africa:_Mkuzi_Game_Reserve-1979) MQNKIPNFNL FFFFLYRMLE IVLATLLGDL QRLRVLTPQQ RAVAFFRANT KELEDFLCSD GQSEEILSGP LLNRLLEPSG PLDILTGYHL 90
Ken05/Tk1_(Kenya-2005) MQNKIPNFNL FFFFLYKMLE IVLATLLGDL QKLKDLTPMQ RAVAFFRANT KELEDFLCPD GQSEELLPGL LLNRLLEPSG PIDILTGYHL 90
Ken06.Bus_(Kenya-2006) MQNKIPNFNL FFFFLYKMLE IVLATLLGDL QQLKDLTPKQ RAVAFFRANT KELEDFLYPD GQTEELLPGF LLNHLLEPSG PIEILTGYHL 90
Malawi_Lil-20/1_(Malawi:_Chalaswa-1983) MQ RAVAFFRVNT KELEDFLYPD GQSEELLPGL LLNRLLEPSG PIDILTGYHL 52
100 120 140 160 180
Ghana2022-62_MGF_360-18R MLER LYDANIYNIL SRLRPEKVRN KAIELYWVFR AIHICHAPLV LDIVRYEEPD FAELAFICAA YFGEPQVMYL 74
200 220 240
Ghana2022-62_MGF_360-18R LYKYMPLTRA VLTDAIRISL ESNNQVGICY AYLMGGSLKG LVSAPLRKRL RAK-LRSQRK KKDVLSPHDF LLLLQ* 149

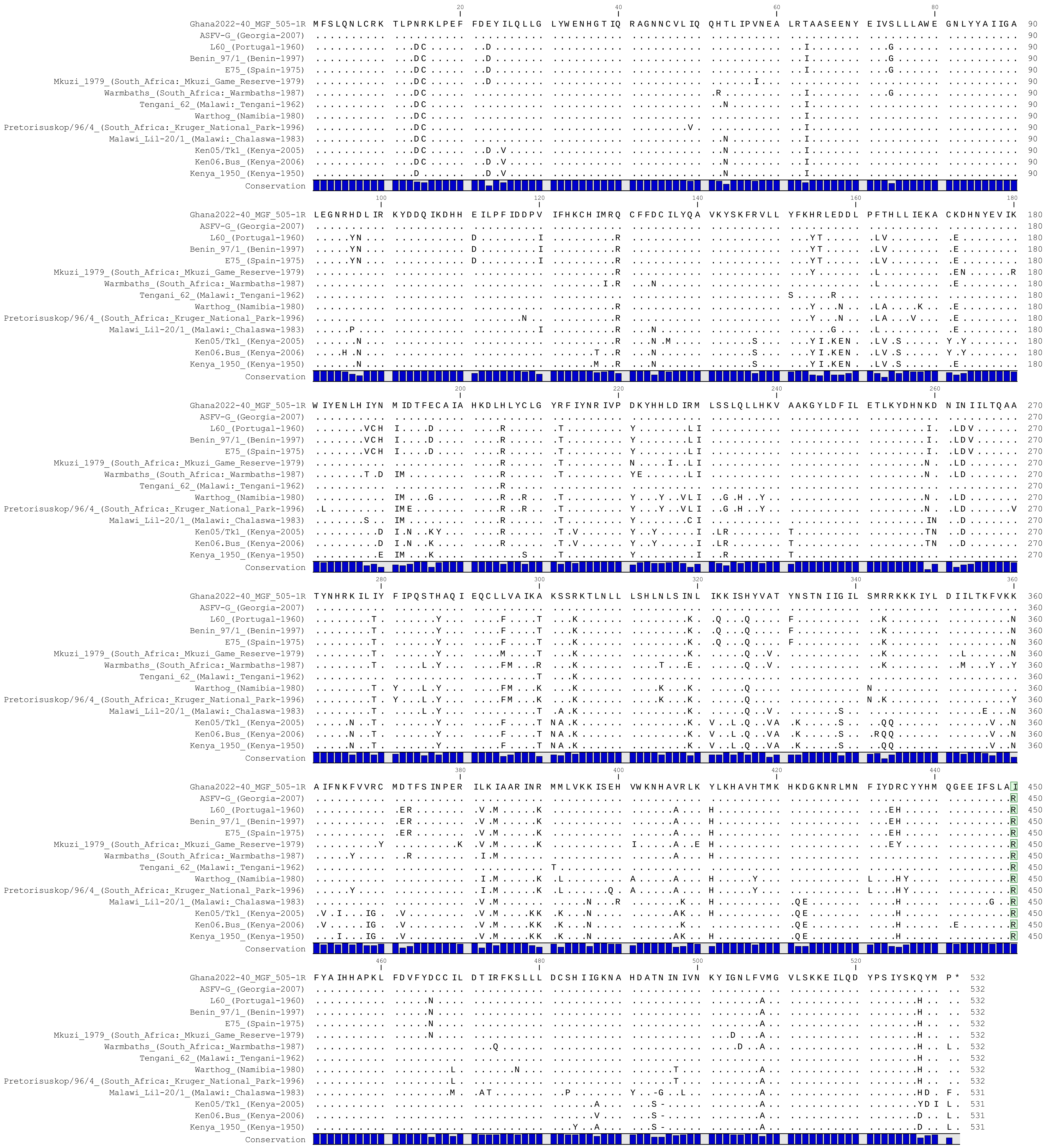

Ghana2022-40_MGF_505-1R
ASFV-G_(Georgia-2007)
L60_(Portugal-1960)
Benin_97/1_(Benin-1997)
E75_(Spain-1975)
Mkuzi_1979_(South_Africa:_Mkuzi_Game_Reserve-1979)
Warmbaths_(South_Africa:_Warmbaths-1987)
Tengani_62_(Malawi:_Tengani-1962)
Warthog_(Namibia-1980)
Pretorisuskop/96/4_(South_Africa:_Kruger_National_Park-1996)
Malawi_Lil-20/1_(Malawi:_Chalaswa-1983)
Ken05/Tk1_(Kenya-2005)
Ken06.Bus_(Kenya-2006)
Kenya_1950_(Kenya-1950)
Conservation
MFSLQNLCRK TLPNRKLPEF FDEYILQLLG LYWENHGTIQ RAGNNCVLIQ QHTLIPVNEA LRTAASEENY EIVSLLLAWE GNLYYAIIGA 90
LEGNRHDLIR KYDDQIKDHH EILPFIDDPV IFHKCHIMRQ CFFDCILYQA VKYSKFRVLL YFKHRLEDDL PFTHLLIEKA CKDHNYEVIK 180
WIYENLHIYN MIDTFECAIA HKDLHLYCLG YRFIYNRIVP DKYHHLDIRM LSSLQLLHKV AAKGYLDFIL ETLKYDHNKD NINIILTQAA 270
TYNHRKILIY FIPQSTHAQI EQCLLVAIKA KSSRKTLNLL LSHLNLSINL IKKISHYVAT YNSTNIIGIL SMRRKKKIYL DIILTKFVKK 360
AIFNKFVVRC MDTFSINPER ILKIAARINR MMLVKKISEH VWKNHAVRLK YLKHAVHTMK HKDGKNRLMN FIYDRCYYHM QGEEIFSLAI 450
FYAIHHAPKL FDVFYDCCIL DTIRFKSLLL DCSHIIGKNA HDATNINIVN KYIGNLFVMG VLSKKEILQD YPSIYSKQYM P* 532

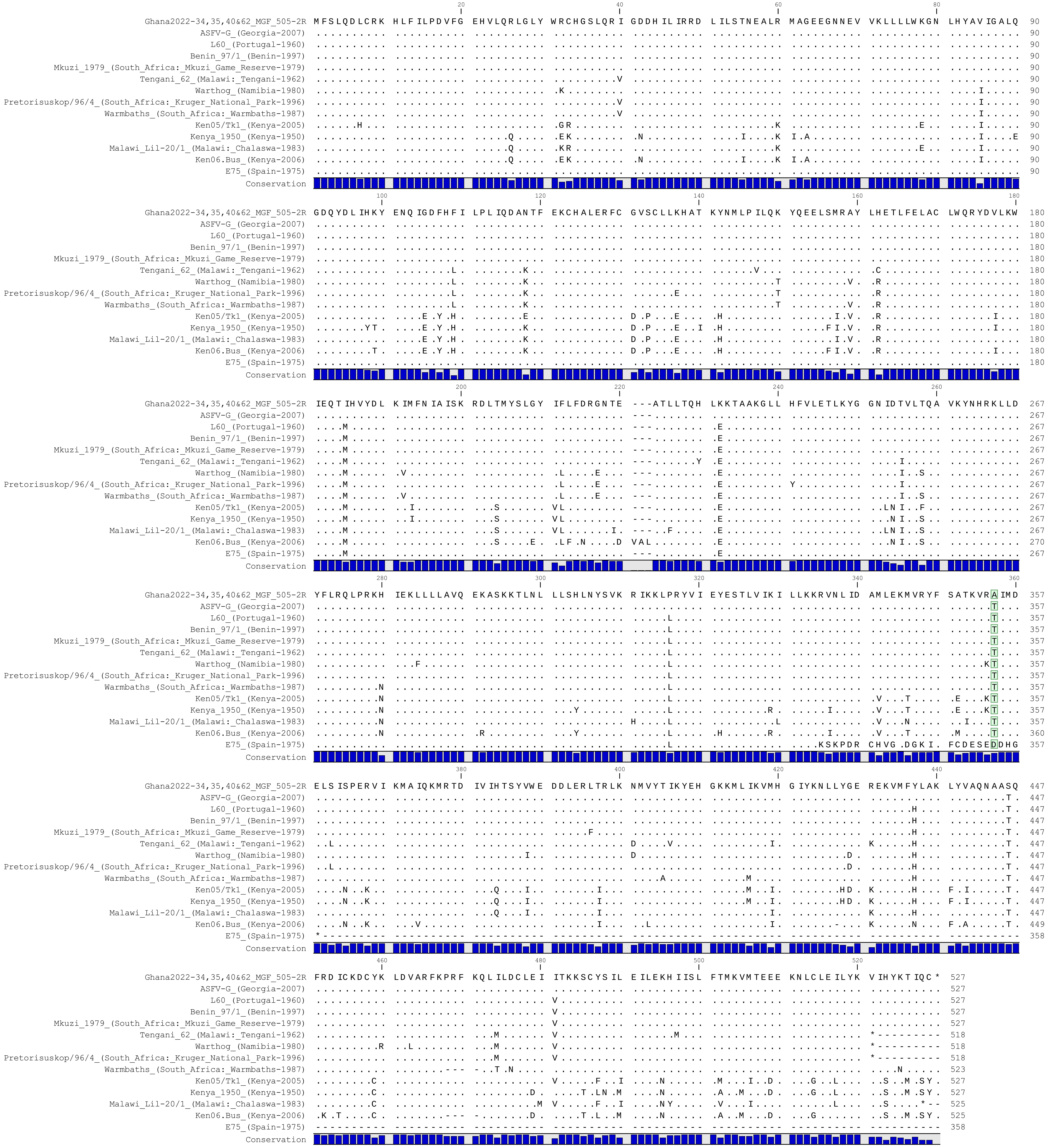
Ghana2022-34,35,40&62_MGF_505-2R
ASFV-G_(Georgia-2007)
L60_(Portugal-1960)
Benin_97/1_(Benin-1997)
Mkuzi_1979_(South_Africa:_Mkuzi_Game_Reserve-1979)
Tengani_62_(Malawi:_Tengani-1962)
Warthog_(Namibia-1980)
Pretorisuskop/96/4_(South_Africa:_Kruger_National_Park-1996)
Warmbaths_(South_Africa:_Warmbaths-1987)
Ken05/Tk1_(Kenya-2005)
Kenya_1950_(Kenya-1950)
Malawi_Lil-20/1_(Malawi:_Chalaswa-1983)
Ken06.Bus_(Kenya-2006)
E75_(Spain-1975)
Conservation
MFSLQDLCRK HLFILPDVFG EHVLQRLGLY WRCHGSLQRI GDDHILIRRD LILSTNEALR MAGEEGNNEV VKLLLLWKGN LHYAVIGALQ 90
GDQYDLIHKY ENQIGDFHFI LPLIQDANTF EKCHALERFC GVSCLLKHAT KYNMLPILQK YQEELSMRAY LHETLFELAC LWQRYDVLKW 180
IEQTIHVYDL KIMFNIAISK RDLTMYSLGY IFLFDRGNTE ---ATLLTQH LKKTAAKGLL HFVLETLKYG GNIDTVLTQA VKYNHRKLLD 267
YFLRQLPRKH IEKLLLLAVQ EKASKKTLNL LLSHLNYSVK RIKKLPRYVI EYESTLVIKI LLKKRVNLID AMLEKMVRYF SATKVRAIMD 357
ELSISPERVI KMAIQKMRTD IVIHTSYVWE DDLERLTRLK NMVYTIKYEH GKKMLIKVMH GIYKNLLYGE REKVMFYLAK LYVAQNAASQ 447
FRDICKDCYK LDVARFKPRF KQLILDCLEI ITKKSCYSIL EILEKHIISL FTMKVMTEEE KNLCLEILYK VIHYKTIQC* 527

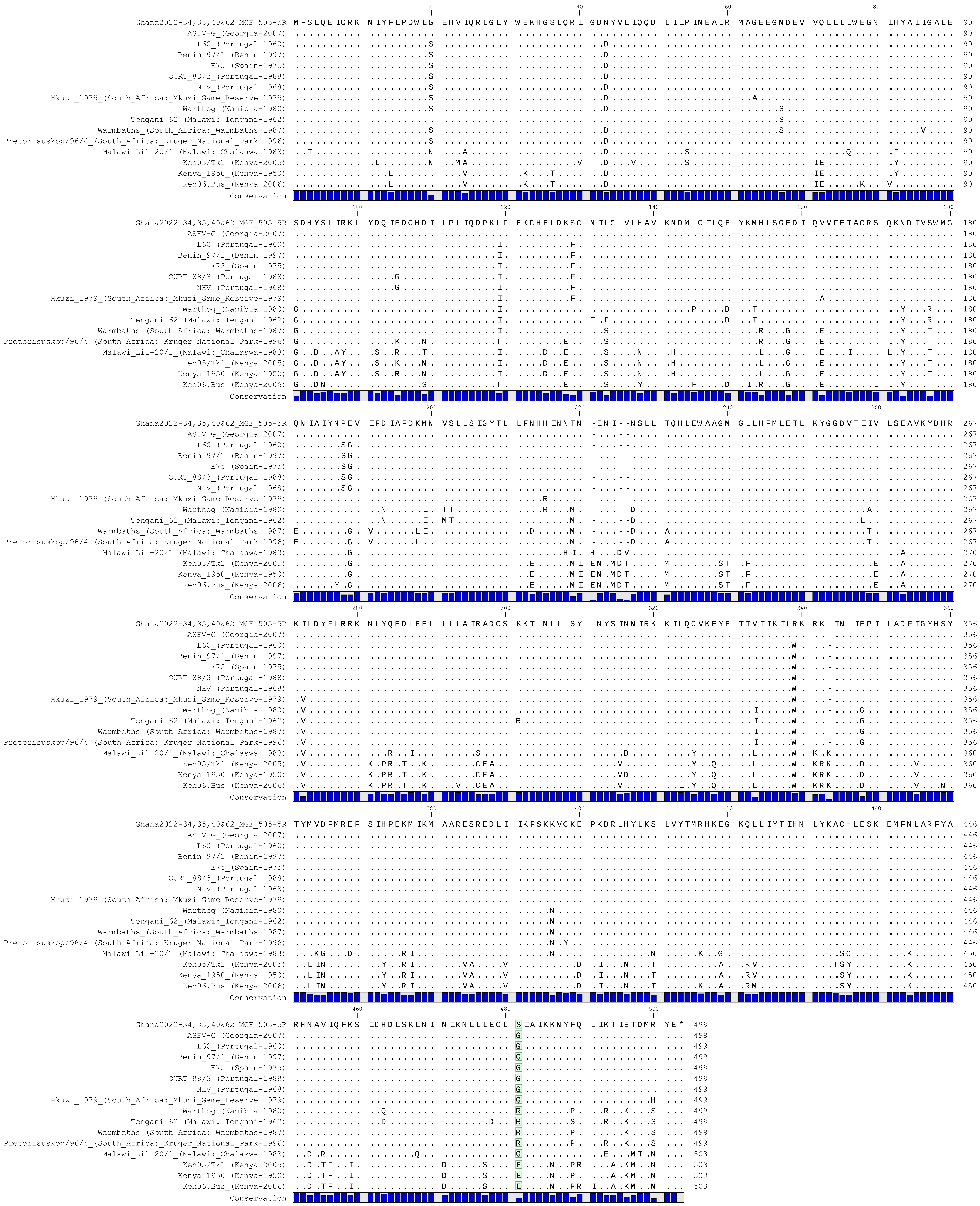
Ghana2022-34,35,40&62_MGF_505-5R
ASFV-G_(Georgia-2007)
L60_(Portugal-1960)
Benin_97/1_(Benin-1997)
E75_(Spain-1975)
OURT_88/3_(Portugal-1988)
NHV_(Portugal-1968)
Mkuzi_1979_(South_Africa:_Mkuzi_Game_Reserve-1979)
Warthog_(Namibia-1980)
Tengani_62_(Malawi:_Tengani-1962)
Warmbaths_(South_Africa:_Warmbaths-1987)
Pretorisuskop/96/4_(South_Africa:_Kruger_National_Park-1996)
Malawi_Lil-20/1_(Malawi:_Chalaswa-1983)
Ken05/Tk1_(Kenya-2005)
Kenya_1950_(Kenya-1950)
Ken06.Bus_(Kenya-2006)
Conservation

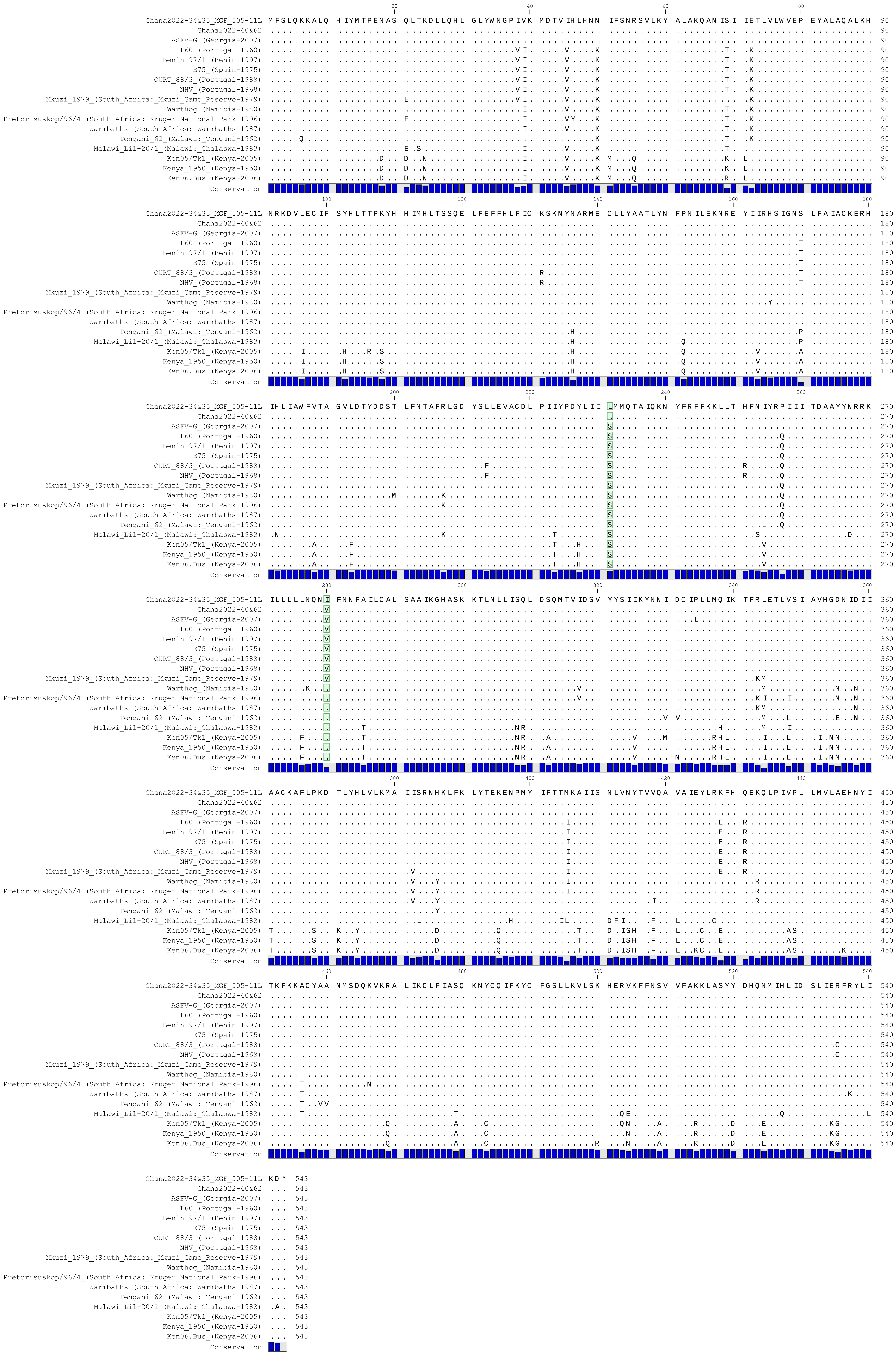

Ghana2022-34&35_MGF_505-11L
Ghana2022-40&62
ASFV-G_(Georgia-2007)
L60_(Portugal-1960)
Benin_97/1_(Benin-1997)
E75_(Spain-1975)
OURT_88/3_(Portugal-1988)
NHV_(Portugal-1968)
Mkuzi_1979_(South_Africa:_Mkuzi_Game_Reserve-1979)
Warthog_(Namibia-1980)
Pretorisuskop/96/4_(South_Africa:_Kruger_National_Park-1996)
Warmbaths_(South_Africa:_Warmbaths-1987)
Tengani_62_(Malawi:_Tengani-1962)
Malawi_Lil-20/1_(Malawi:_Chalaswa-1983)
Ken05/Tk1_(Kenya-2005)
Kenya_1950_(Kenya-1950)
Ken06.Bus_(Kenya-2006)
Conservation
MFSLQKKALQ HIYMTPENAS QLTKDLLQHL GLYWNGPIVK MDTVIHLHNN IFSNRSVLKY ALAKQANISI IETLVLWVEP EYALAQALKH 90
NRKDVLECIF SYHLTTPKYH HIMHLTSSQE LFEFFHLFIC KSKNYNARME CLLYAATLYN FPNILEKNRE YIIRHSIGNS LFAIACKERH 180
IHLIAWFVTA GVLDTYDDST LFNTAFRLGD YSLLEVACDL PIIYPDYLII LMMQTAIQKN YFRFFKKLLT HFNIYRPIII TDAAYYNRRK 270
ILLLLLNQNI FNNFAILCAL SAAIKGHASK KTLNLLISQL DSQMTVIDSV YYSIIKYNNI DCIPLLMQIK TFRLETLVSI AVHGDNIDII 360
AACKAFLPKD TLYHLVLKMA IISRNHKLFK LYTEKENPMY IFTTMKAIIS NLVNYTVVQA VAIEYLRKFH QEKQLPIVPL LMVLAEHNYI 450
TKFKKACYAA NMSDQKVKRA LIKCLFIASQ KNYCQIFKYC FGSLLKVLSK HERVKFFNSV VFAKKLASYY DHQNMIHLID SLIERFRYLI 540
KD* 543

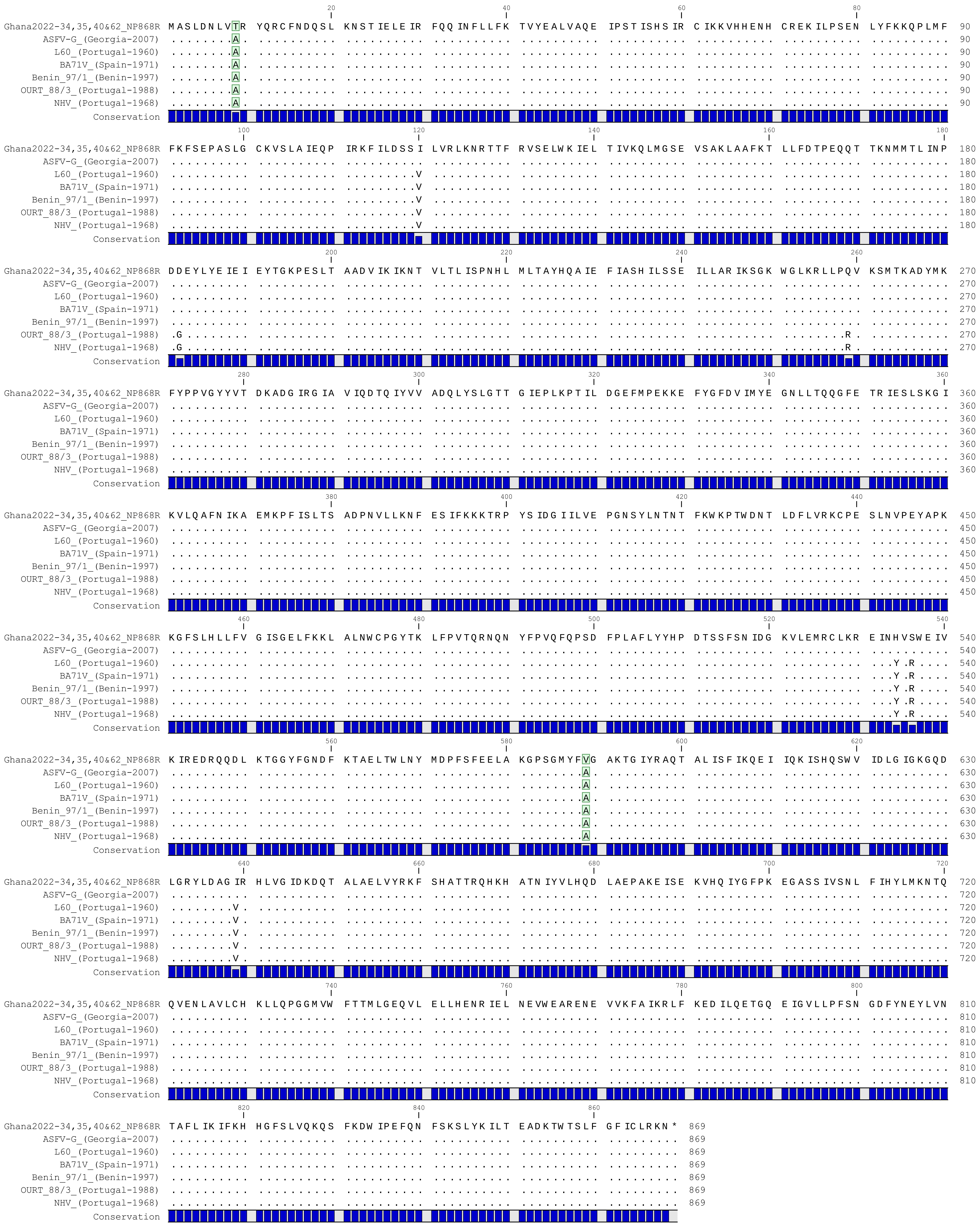

Ghana2022-34,35,40&62_NP868R MASLDNLVTR YQRCFNDQSL KNSTIELEIR FQQINFLLFK TVYEALVAQE IPSTISHSIR CIKKVHHENH CREKILPSEN LYFKKQPLMF 90
ASFV-G_(Georgia-2007) ....A. 90
L60_(Portugal-1960) ....A. 90
BA71V_(Spain-1971) ....A. 90
Benin_97/1_(Benin-1997) ....A. 90
OURT_88/3_(Portugal-1988) ....A. 90
NHV_(Portugal-1968) ....A. 90
Conservation
Ghana2022-34,35,40&62_NP868R FKFSEPASLG CKVSLAIEQP IRKFILDSSI LVRLKNRTTF RVSELWKIEL TIVKQLMGSE VSAKLAAFKT LLFDTPEQQT TKNMMTLINP 180
ASFV-G_(Georgia-2007) 180
L60_(Portugal-1960) ....V 180
BA71V_(Spain-1971) ....V 180
Benin_97/1_(Benin-1997) ....V 180
OURT_88/3_(Portugal-1988) ....V 180
NHV_(Portugal-1968) ....V 180
Conservation
Ghana2022-34,35,40&62_NP868R DDEYLYEIEI EYTGKPESLT AADVIKIKNT VLTLISPNHL MLTAYHQAIE FIASHILSSE ILLARIKSGK WGLKRLLPQV KSMTKADYMK 270
ASFV-G_(Georgia-2007) 270
L60_(Portugal-1960) 270
BA71V_(Spain-1971) 270
Benin_97/1_(Benin-1997) 270
OURT_88/3_(Portugal-1988) .G........ ....R 270
NHV_(Portugal-1968) .G........ ....R 270
Conservation
Ghana2022-34,35,40&62_NP868R FYPPVGYYVT DKADGIRGIA VIQDTQIYVV ADQLYSLGTT GIEPLKPTIL DGEFMPEKKE FYGFDVIMYE GNLLTQQGFE TRIESLSKGI 360
ASFV-G_(Georgia-2007) 360
L60_(Portugal-1960) 360
BA71V_(Spain-1971) 360
Benin_97/1_(Benin-1997) 360
OURT_88/3_(Portugal-1988) 360
NHV_(Portugal-1968) 360
Conservation
Ghana2022-34,35,40&62_NP868R KVLQAFNIKA EMKPFISLTS ADPNVLLKNF ESIFKKKTRP YSIDGIILVE PGNSYLNTNT FKWKPTWDNT LDFLVRKCPE SLNVPEYAPK 450
ASFV-G_(Georgia-2007) 450
L60_(Portugal-1960) 450
BA71V_(Spain-1971) 450
Benin_97/1_(Benin-1997) 450
OURT_88/3_(Portugal-1988) 450
NHV_(Portugal-1968) 450
Conservation
Ghana2022-34,35,40&62_NP868R KGFSLHLLFV GISGELFKKL ALNWCPGYTK LFPVTQRNQN YFPVQFQPSD FPLAFLYYHP DTSSFSNIDG KVLEMRCLKR EINHVSWEIV 540
ASFV-G_(Georgia-2007) 540
L60_(Portugal-1960) ....Y.R.... 540
BA71V_(Spain-1971) ....Y.R.... 540
Benin_97/1_(Benin-1997) ....Y.R.... 540
OURT_88/3_(Portugal-1988) ....Y.R.... 540
NHV_(Portugal-1968) ....Y.R.... 540
Conservation
Ghana2022-34,35,40&62_NP868R KIREDRQQDL KTGGYFGNDF KTAELTWLNY MDPFSFEELA KGPSGMYFVG AKTGIYRAQT ALISFIKQEI IQKISHQSWV IDLGIGKGQD 630
ASFV-G_(Georgia-2007) ....A. 630
L60_(Portugal-1960) ....A. 630
BA71V_(Spain-1971) ....A. 630
Benin_97/1_(Benin-1997) ....A. 630
OURT_88/3_(Portugal-1988) ....A. 630
NHV_(Portugal-1968) ....A. 630
Conservation
Ghana2022-34,35,40&62_NP868R LGRYLDAGIR HLVGIDKDQT ALAELVYRKF SHATTRQHKH ATNIYVLHQD LAEPAKEISE KVHQIYGFPK EGASSIVSNL FIHYLMKNTQ 720
ASFV-G_(Georgia-2007) 720
L60_(Portugal-1960) ....V 720
BA71V_(Spain-1971) ....V 720
Benin_97/1_(Benin-1997) ....V 720
OURT_88/3_(Portugal-1988) ....V 720
NHV_(Portugal-1968) ....V 720
Conservation
Ghana2022-34,35,40&62_NP868R QVENLAVLCH KLLQPGGMVW FTTMLGEQVL ELLHENRIEL NEVWEARENE VVKFAIKRLF KEDILQETGQ EIGVLLPFSN GDFYNEYLVN 810
ASFV-G_(Georgia-2007) 810
L60_(Portugal-1960) 810
BA71V_(Spain-1971) 810
Benin_97/1_(Benin-1997) 810
OURT_88/3_(Portugal-1988) 810
NHV_(Portugal-1968) 810
Conservation
Ghana2022-34,35,40&62_NP868R TAFLIKIFKH HGFSLVQKQS FKDWIPEFQN FSKSLYKILT EADKTWTSLF GFICLRKN* 869
ASFV-G_(Georgia-2007) 869
L60_(Portugal-1960) 869
BA71V_(Spain-1971) 869
Benin_97/1_(Benin-1997) 869
OURT_88/3_(Portugal-1988) 869
NHV_(Portugal-1968) 869
Conservation

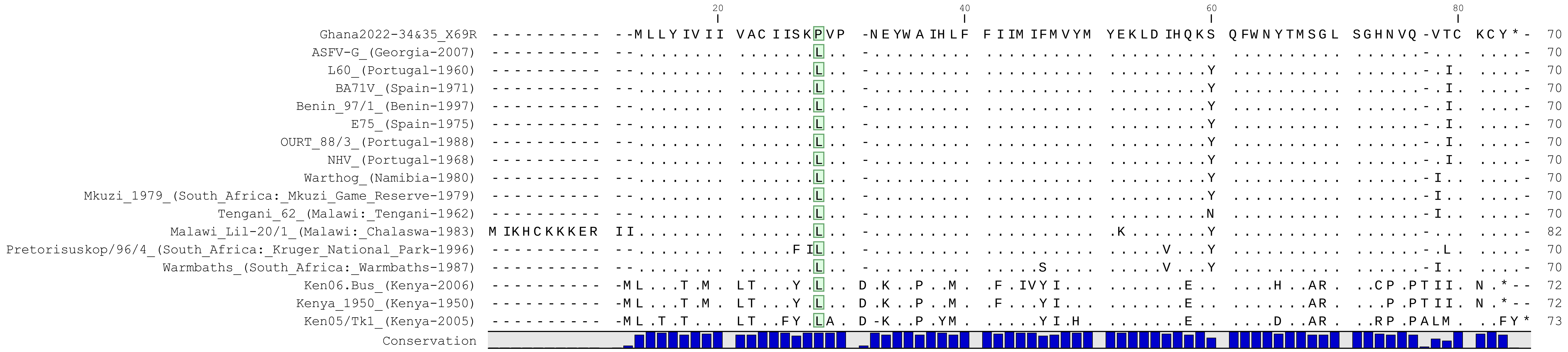

20
40
60
80
Ghana2022-34&35_X69R ------------ --MLLYIVII VACIISKPVP -NEYWAIHLF FIIMIFMVYM YEKLDIHQKS QFWNYTMSGL SGHNVQ-VTC KCY*- 70
ASFV-G_(Georgia-2007) 70
L60_(Portugal-1960) 70
BA71V_(Spain-1971) 70
Benin_97/1_(Benin-1997) 70
E75_(Spain-1975) 70
OURT_88/3_(Portugal-1988) 70
NHV_(Portugal-1968) 70
Warthog_(Namibia-1980) 70
Mkuzi_1979_(South_Africa:_Mkuzi_Game_Reserve-1979) 70
Tengani_62_(Malawi:_Tengani-1962) 70
Malawi_Lil-20/1_(Malawi:_Chalaswa-1983) 82
Pretorisuskop/96/4_(South_Africa:_Kruger_National_Park-1996) 70
Warmbaths_(South_Africa:_Warmbaths-1987) 70
Ken06.Bus_(Kenya-2006) 72
Kenya_1950_(Kenya-1950) 72
Ken05/Tk1_(Kenya-2005) 73
Conservation
